# Single-Axis Rotational Inertial Navigation Systems for USVs: A Review of Key Technologies

**DOI:** 10.3390/mi17050557

**Published:** 2026-04-30

**Authors:** Enqing Su, Junwei Wang, Weijie Sheng, Yi Mou, Teng Li, Jianguo Liu

**Affiliations:** 1 School of Electrical and Electronic Engineering, Wuhan Polytechnic University, Wuhan 430048, China; suenqingsu@gmail.com (E.S.); mouyi@whpu.edu.cn (Y.M.); liteng013@whpu.edu.cn (T.L.); 2 School of Artificial Intelligence and Automation, Hohai University, Nanjing 210098, China; jw_wang@hhu.edu.cn; 3 School of Information Engineering, Yangzhou University, Yangzhou 225127, China; wjsheng@yzu.edu.cn

**Keywords:** unmanned surface vehicle (USV), global navigation satellite system (GNSS), single-axis rotational inertial navigation system (SRINS), micro-electro-mechanical system (MEMS)

## Abstract

In complex marine environments, achieving low-cost, highly reliable, and continuous navigation is crucial for the intelligent and autonomous operation of unmanned surface vehicles (USVs). Currently, the integrated Global Navigation Satellite System and Strapdown Inertial Navigation System (GNSS/SINS) serves as the primary navigation architecture for USVs. While the cost of high-performance GNSS receivers has steadily decreased, high-precision SINS remains prohibitively expensive. Consequently, micro-electromechanical system (MEMS)-based SINS has emerged as a preferred alternative due to its favorable balance of cost, power consumption, and size. However, significant inertial sensor errors make it difficult to maintain high-precision positioning during GNSS outages. To address this limitation, the single-axis rotational inertial navigation system (SRINS) has been introduced. Nevertheless, constrained by the single-axis mechanical structure and complex sea state disturbances, the system still struggles to effectively modulate random errors and azimuth gyroscope drift, rendering it insufficient for highly demanding navigation tasks. To overcome these bottlenecks, this article systematically reviews four core technologies: (1) Comprehensive denoising and temperature drift compensation techniques for MEMS gyroscopes; (2) rapid moving-base initial alignment models under high sea state disturbances; (3) fast online calibration methods for azimuth gyroscope drift; and (4) adaptive and robust GNSS/SINS integration architectures capable of accommodating high-dynamic conditions and non-Gaussian interference. Finally, this article discusses the engineering conflict between deploying high-precision algorithms and the limited onboard computational capacity of USVs. It concludes by highlighting a highly promising navigation paradigm for future research: the integration of factor graph optimization with physics-informed deep learning.

## 1. Introduction

Driven by the expansion of the blue economy and the growing need to safeguard maritime interests, unmanned surface vehicles (USVs) are transitioning from traditional remote-controlled systems into intelligent, autonomous operational platforms [1,2]. The advanced autonomy of USVs—encompassing environmental perception, path planning, and multi-vessel collaboration—heavily depends on continuous, accurate, and stable position and attitude information. Currently, the integrated Global Navigation Satellite System and Strapdown Inertial Navigation System (GNSS/SINS) serves as the foundational architecture for USVs to acquire high-precision positioning. However, in complex or extreme marine environments, GNSS signals are highly susceptible to electromagnetic interference and physical obstruction, frequently resulting in signal degradation or complete outages. Without external measurement updates, the inherent errors of the SINS accumulate rapidly over time, leading to severe errors in position and attitude outputs. Because USVs are frequently required to operate in such GNSS-denied conditions, the development of highly reliable, interference-resistant autonomous navigation systems remains a paramount engineering challenge.

With the development of USVs toward lightweight and intelligent designs, onboard equipment faces spatial and power constraints. Although low-cost micro-electro-mechanical system (MEMS)-integrated navigation schemes provide a viable alternative, they inherently suffer from bias drift and stochastic noise [3]. Conventional algorithms, such as the Kalman filter (KF), struggle to eliminate these hardware errors at the physical level [4]. To address this limitation, rotation modulation technology (RMT) has been introduced to fundamentally suppress constant sensor errors [5]. By periodically rotating the inertial sensors, RMT converts constant drift into periodic signals. Integrating these modulated signals over a full rotation cycle effectively counteracts the constant drift. Rotating inertial navigation systems are primarily classified into single-axis, dual-axis, and triaxial configurations. While dual-axis and triaxial systems effectively modulate constant drifts along all three axes, they exhibit larger volumes, greater structural complexity, and higher manufacturing costs; conversely, single-axis rotation systems offer the advantages of lower cost, compact size, and simpler structure while modulating constant drifts perpendicular to the rotation axis [6,7]. For MEMS-SINS, the single-axis configuration demonstrates superior suitability for USV navigation applications, considering development cost and complexity. However, this technology cannot autonomously compensate for constant gyroscope drift along the rotation axis and provides no inherent suppression of gyroscope random errors or temperature drift, necessitating dedicated compensation methodologies.

Modern small USVs typically cruise at 20–50 knots with a maximum speed of 80 knots, requiring continuous operational capability in sea states 4–5 [1]. To rapidly deploy small USVs, initial alignment of the inertial system must first be completed. As the prerequisite for SINS navigation and positioning, initial alignment exhibits strongly nonlinear characteristics under complex sea conditions due to environmental disturbances, compounded by significant device errors in low-cost SINS [2]. Traditional optimal estimation-based alignment methods suffer from prolonged duration and reduced accuracy; therefore, nonlinear rapid initial alignment models for complex sea states require investigation. Following initial alignment, the system enters the navigation state. While rotation modulation technology enables low-cost GNSS/SINS-integrated navigation systems to achieve continuous, high-precision positioning for USVs, aggressive maneuvers (e.g., sharp turns, circling, acceleration/deceleration, high-speed navigation) during complex operations induce filter model errors [5]. Concurrently, GNSS signals are susceptible to interference from waves, currents, obstructions, and electromagnetic factors. To mitigate the integrated navigation system’s sensitivity to vessel dynamics and abnormal GNSS signals, research on navigation algorithms with enhanced adaptability and robustness is imperative.

While MEMS-SRINS presents significant application potential for small USVs, the existing review literature has not systematically addressed the engineering constraints caused by single-axis structural limitations and sea state disturbances. Current USV reviews primarily focus on macroscopic systems or high-level algorithms. One category of research concentrates on comprehensive applications and system platforms, such as communication networking, multi-vessel collaboration, disaster management, and bathymetric surveys [8,9,10,11,12]. Another category focuses on motion control and planning, including global route planning, obstacle avoidance, and path-following control [13,14]. These studies typically treat the navigation system as an ideal module capable of providing precise state estimation, rarely delving into the error suppression and environmental adaptability of underlying inertial sensors under complex marine conditions. Consequently, there is a lack of technical depth regarding the physical-level error characteristics of inertial units in the USV field.

In addition, reviews targeting inertial navigation and positioning technologies—such as information-aided navigation architectures or deep learning-based inertial positioning methods—generally cater to generic platforms like pedestrians, drones, or land vehicles [15,16]. These studies seldom consider the high-frequency hull dynamics of USVs induced by wave disturbances or the specific mechanical limitations of single-axis rotation systems, which inherently fail to modulate azimuth drift. Consequently, a distinct research gap exists between macroscopic USV application reviews and general inertial navigation algorithm reviews. There is currently a lack of a dedicated review examining SRINS for USVs to address practical engineering challenges, including single-axis error modulation, dynamic alignment under complex marine conditions, and the high-precision positioning. To bridge this gap and establish a systematic theoretical reference, this paper conducts a targeted analysis of core technologies. The specific literature search strategy and selection process are detailed as follows.

To ensure a comprehensive and objective analysis of this specific domain, a systematic literature search was conducted to underpin this review. The primary databases utilized included IEEE Xplore, ScienceDirect, Web of Science, and Google Scholar. The search queries were formulated using combinations of keywords such as “unmanned surface vehicle”, “MEMS gyroscope”, “single-axis rotation”, “GNSS/SINS integration”, and “robust filtering”. The literature search covered a broad time window from 1992 to 2026, capturing both the fundamental theories of observability and the latest advancements in deep learning and factor graph optimization. During the screening process, the following inclusion criteria were strictly applied: (1) peer-reviewed journal articles and high-quality international conference proceedings; (2) studies specifically addressing low-cost MEMS characteristics or marine dynamic environments; and (3) selected literature was ultimately evaluated based on methodological innovation, experimental reliability, and citation impact, ensuring that the cited works represent the state-of-the-art developments in this field.

To bridge this gap and further explore the application potential of MEMS-SRINS, this article presents a study on the key algorithms of low-cost integrated navigation systems in complex marine environments. This article focuses on the following aspects: Section 2 introduces the fundamental principles of MEMS-SRINS. Section 3 systematically investigates MEMS gyroscope error compensation technologies. Section 4 discusses rapid initial alignment models under complex sea states. Section 5 presents rapid online calibration methods for azimuth gyroscope drift specific to the single-axis structure. Section 6 analyzes adaptive robust GNSS/SINS algorithms capable of operating under high-dynamic interference. Section 7 discusses the current engineering challenges and outlines future technological trends. Finally, Section 8 provides the concluding remarks. By evaluating both physical-layer modulation and algorithmic compensation, this article aims to overcome the multiple constraints imposed by low-cost hardware, thereby providing a highly reliable and high-precision autonomous navigation paradigm for small USVs.

## 2. Fundamentals

The core advantage of SRINS lies in using physical rotation to modulate the propagation of inherent sensor errors. To provide rigorous theoretical support for the subsequent sections, this section systematically elaborates on the fundamental theory of SRINS. First, the spatial reference frames involved in the system and their coordinate transformation matrices are defined, which serve as the prerequisite for establishing the kinematic model. Based on this spatial structure, the physical mechanism of error modulation induced by single-axis rotation is thoroughly analyzed, and its inherent limitations in low-cost MEMS applications are objectively discussed, thereby laying the foundation for the subsequent review of key technologies.

### 2.1. Coordinate Transformations and Reference Frames

In SRINS, the derivation of navigation solutions and error modulation algorithms relies heavily on the precise definition of spatial reference frames. To accurately describe the vehicle motion and the relative motion introduced by the rotation mechanism, this article adopts standard coordinate system definitions widely used in the literature [17]. The fundamental coordinate frames and their transformation relationships involved in the subsequent algorithm derivations are defined as follows:

1. Earth-centered inertial frame ( i -frame):

The i -frame originates at the Earth’s center. The $x_{i}$ -axis points toward the vernal equinox, the $z_{i}$ -axis aligns with the Earth’s rotation axis pointing toward the North Pole, and the $y_{i}$ -axis completes the right-handed orthogonal coordinate system with the $x_{i}$ and $z_{i}$ axes. This frame serves as the fundamental reference for Newtonian mechanics, and the measurements from inertial sensors (gyroscopes and accelerometers) are referenced to the motion relative to the i -frame.

2. Earth-fixed frame ( e -frame):

The e -frame also originates at the Earth’s center but is fixed to the Earth, rotating with it at an angular velocity $\omega_{ie}$ relative to the i -frame. The $x_{e}$ -axis passes through the intersection of the prime meridian and the equator, and the $z_{e}$ -axis coincides with the $z_{i}$ -axis.

3. Navigation frame ( n -frame):

The n -frame is the local reference coordinate system for navigation calculations. This article employs the East-North-Up geographic coordinate system as the navigation frame. Its origin is located at the vehicle’s center of mass, with the $x_{n}$ , $y_{n}$ , and $z_{n}$ axes pointing in the local East, North, and Up directions, respectively.

4. Body frame ( b -frame):

The b -frame is fixed to the vehicle, with its origin at the center of mass. Conventionally, the $x_{b}$ , $y_{b}$ , and $z_{b}$ axes are defined to point in the right, forward and up directions of the vehicle, respectively. In traditional SINS, the inertial measurement unit (IMU) is usually assumed to be perfectly aligned with the b -frame.

5. Sensor/IMU frame ( s -frame):

This is the core coordinate system distinguishing rotation modulation systems from traditional strapdown systems. The s -frame is fixed to the inertial sensor assembly containing the orthogonal gyroscopes and accelerometers. In SINS, the s -frame undergoes periodic angular position changes relative to the b -frame, driven by the rotation mechanism [18].

6. Coordinate transformations:

In the multi-frame framework, the projection of vectors between different coordinate systems is typically implemented using the direction cosine matrix or quaternions. Taking the direction cosine matrix as an example, if installation errors are neglected, the attitude matrix of the rotational modulation system at any given instant can be decomposed into the product of the vehicle attitude matrix and the relative motion matrix of the rotation mechanism [19]:

(1) $$C_{b}^{n}=C_{s}^{n}C_{b}^{s}$$

where $C_{b}^{n}$ represents the vehicle’s attitude matrix relative to the navigation frame (containing heading, pitch, and roll information), and $C_{b}^{s}$ is the core transformation matrix introduced by the single-axis rotation.

Assuming the system adopts a single-axis rotation scheme around the z -axis (azimuth axis), and the instantaneous rotation angle relative to the b -frame is ϕ , the transformation matrix from the s -frame to the b -frame can be rigorously expressed as:

(2) $$C_{b}^{s}={\left(C_{s}^{b}\right)}^{T}=\left[\begin{array}{ccc} \cos\phi & \sin\phi & 0\\ -\sin\phi & \cos\phi & 0\\ 0 & 0 & 1 \end{array}\right]$$

**Figure 1:**
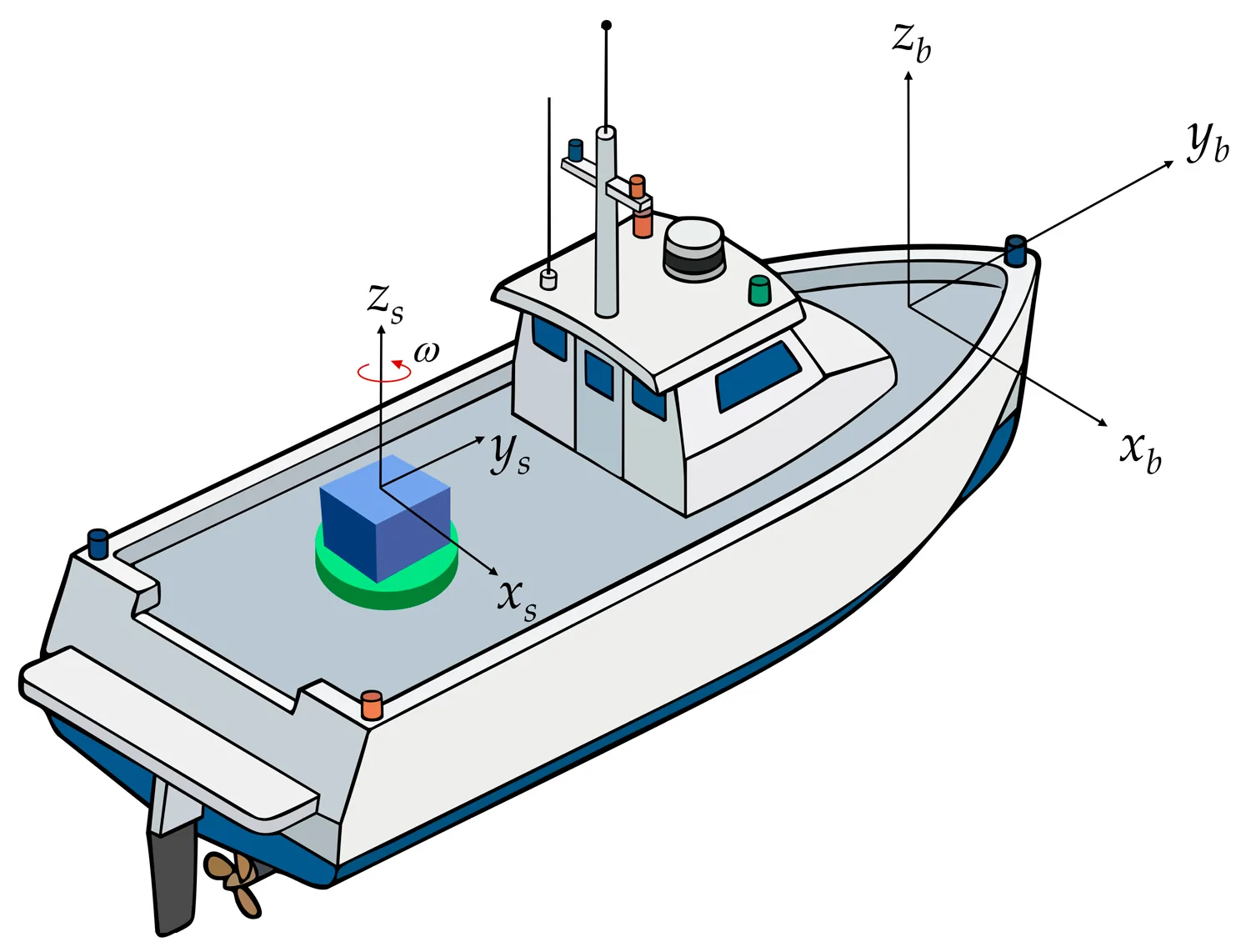
Coordinate frames and mechanical configuration. The body frame ( b -frame: $x_{b}$ , $y_{b}$ , $z_{b}$ ) is aligned with the USV’s right, forward, and up directions; the sensor frame ( s -frame: $x_{s}$ , $y_{s}$ , $z_{s}$ ) represents the axes of the rotating IMU. The IMU rotates around the $z_{s}$ -axis at a rotational angular velocity ω .

To more intuitively illustrate the spatial topological relationships between the aforementioned reference frames and the relative motion introduced by the single-axis rotation mechanism, Figure 1 presents the coordinate system definitions and mechanical architecture of a typical SRINS. Controlling the rotation rate ϕ (i.e., the rotational angular velocity ω ) alters the transformation matrix. This process modulates the IMU’s constant drift into high-frequency periodic signals, allowing subsequent filters to remove the errors. This constitutes the physical essence of various rotation scheme designs [18].

### 2.2. Error Modulation Mechanisms

Based on the coordinate transformations discussed in the previous section, the core physical significance of rotation modulation lies in actively changing the projection characteristics of the sensor’s constant drift within the navigation frame by introducing relative rotational motion.

- Classical Modulation Mechanism and Limitations

Assume that the constant drift of the inertial sensor in the s -frame is $\varepsilon^{s}$ . In the ideal state of single-axis constant-speed rotation around the z -axis with an angular velocity ω , the equivalent projection of this error in the vehicle frame ( b -frame) can be derived as [20]:

(3) $$\varepsilon^{b}=C_{s}^{b}\varepsilon^{s}=\left[\begin{array}{ccc} \cos\omega t & -\sin\omega t & 0\\ \sin\omega t & \cos\omega t & 0\\ 0 & 0 & 1 \end{array}\right]\left[\begin{array}{c} \varepsilon_{x}\\ \varepsilon_{y}\\ \varepsilon_{z} \end{array}\right]$$

Similarly, assuming the constant bias of the accelerometer is $\nabla^{s}$ , its equivalent projection can be expressed as:

(4) $$\nabla^{b}=C_{s}^{b}\nabla^{s}=\left[\begin{array}{ccc} \cos\omega t & -\sin\omega t & 0\\ \sin\omega t & \cos\omega t & 0\\ 0 & 0 & 1 \end{array}\right]\left[\begin{array}{c} \nabla_{x}\\ \nabla_{y}\\ \nabla_{z} \end{array}\right]$$

**Figure 2:**
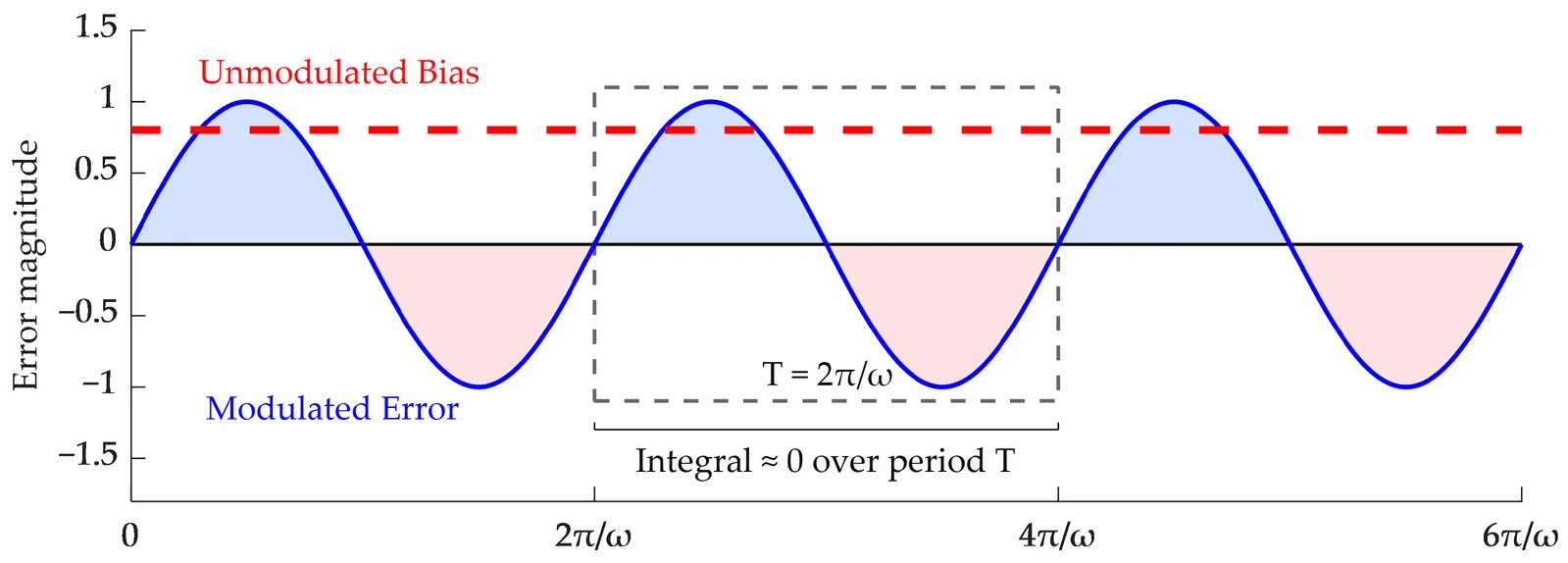
Modulation of constant drift perpendicular to the rotation axis.

**Figure 3:**
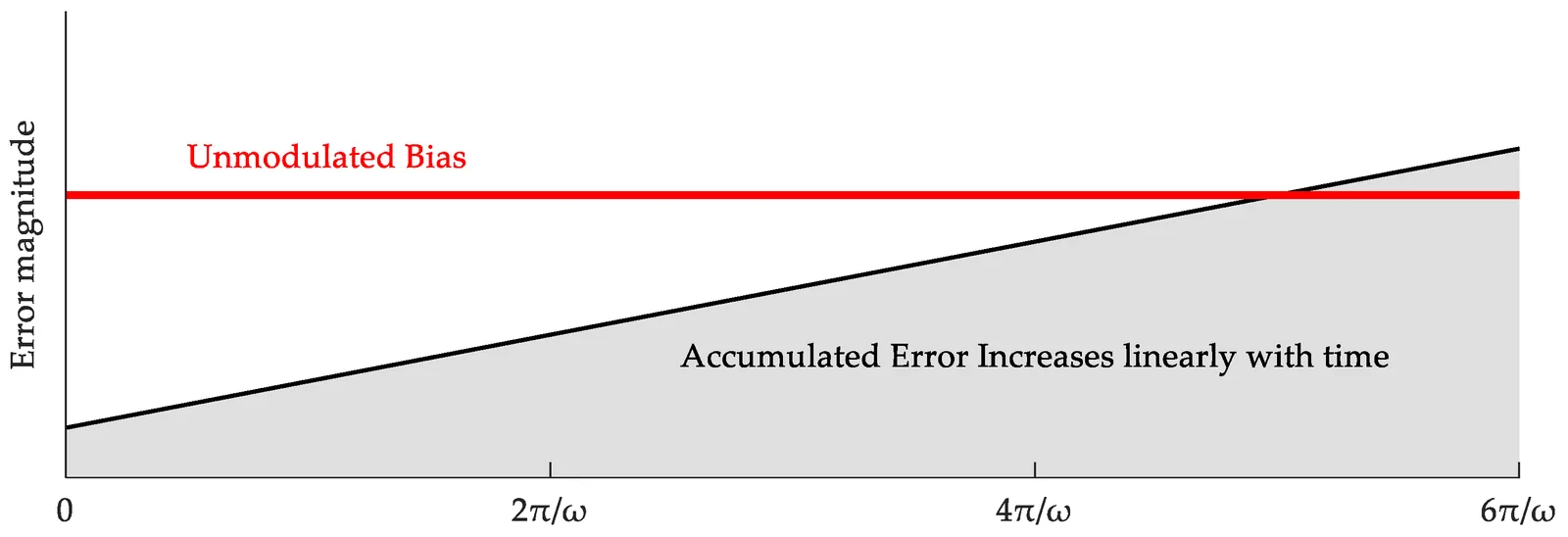
Linear accumulation of unmodulated drift parallel to the rotation axis.

As illustrated by the time-domain modulation waveform in Figure 2 and Figure 3, Equation (3) indicates that the constant drift of the gyroscope perpendicular to the rotation axis ( $\varepsilon_{x}$ , $\varepsilon_{y}$ ) is modulated into periodic signals, which tend to zero when integrated over a full cycle [21,22]. However, this mechanism has two inherent limitations: first, the error parallel to the rotation axis ( $\varepsilon_{z}$ ) cannot be modulated; second, long-term slow-varying non-stationary errors will still accumulate severely in the traditional rotation-stop scheme [23]. The constant drift of the accelerometer exhibits similar modulation characteristics, which will not be further detailed here.

- Rotation-Induced Errors

In addition to the inherent theoretical limitations of the aforementioned classical mechanism, applying low-cost MEMS to USVs under complex sea states also excites secondary errors induced by single-axis rotation. The ideal modulation in (3) and (4) relies on the assumption that the inertial sensor errors are constant and the base is relatively stable. However, random noise and temperature drift in MEMS gyroscopes violate this assumption. As a result, rotation causes severe spectral aliasing of these non-constant errors. Furthermore, the severe sway of the USV induced by waves is superimposed on the internal single-axis rotation of the system. This dual dynamic coupling amplifies parasitic errors, such as scale factor asymmetry [22]. It also distorts or diverges from traditional static alignment and linear filtering models.

In summary, purely physical rotation modulation cannot address the nonlinear challenges introduced by complex marine environments and low-cost hardware. The system requires in-depth error compensation and model reconstruction at the algorithmic level, which constitutes the physical motivation for the subsequent discussion of the related core technologies in this article.

## 3. MEMS Gyroscope Error Compensation

Restricted by the manufacturing technology of MEMS-SINS, the raw measurement signals of MEMS gyroscopes are contaminated by significant random noise and temperature drift. As stated in the theoretical fundamentals in Section 2 of this paper, the physical modulation mechanism of SRINS heavily relies on an ideal assumption: the drift of inertial sensors remains constant or varies slowly within a rotation period. Small USVs frequently navigate in harsh marine environments. Under such working conditions, the high-frequency broadband noise and low-frequency temperature hysteresis effect of MEMS devices violate this ideal physical premise. This not only makes it impossible to fully integrate and eliminate time-varying drift via mechanical rotation, but also triggers additional nonlinear coupling issues. Therefore, constrained by the hardware conditions of small USVs, breaking through hardware performance bottlenecks and adopting algorithm-driven error compensation mechanisms have become effective means to guarantee the rotation modulation performance of SRINS and improve navigation accuracy. From the two dimensions of stochastic error denoising and temperature drift compensation, this section reviews the relevant key technologies.

### 3.1. Stochastic Error Denoising

During measurement, low-cost MEMS gyroscopes are frequently corrupted by complex random errors, including quantization noise, angle random walk, and bias instability. For the SRINS, the precise suppression of random noise is not merely traditional signal smoothing, but an essential procedure to guarantee the performance of the modulation mechanism. If left unfiltered, these non-stationary broadband noise signals alias with the micro-motor’s rotation frequency. This aliasing obscures the weak true motion signals extracted during rotation.

In addressing such errors deeply coupled with rotation, traditional high-precision optical systems, such as RLGs, typically rely on dedicated physical compensation models to eliminate drift induced by rotation and reversal, whereas for the significant random drift of MEMS, recent studies have proposed introducing a multi-IMU array at the hardware level to achieve deep physical synergy between array denoising and single-axis rotation on the power spectrum [24,25]. However, constrained by the physical limitations of small USVs, schemes that rely on expensive optical devices or hardware redundancy are difficult to deploy at scale.

Therefore, exploring denoising strategies at the algorithmic level has become a viable approach. Denoising strategies for random errors have shifted from traditional statistical filtering to data-driven deep learning. Currently, this field is moving toward a hybrid paradigm that combines physical priors with data-driven models. However, a primary limitation of this field in current USV applications is that wave slamming and multi-degree-of-freedom hull vibrations excite strong non-Gaussian and broadband impulsive noise. From a hydrodynamic perspective, surface vessels operating in complex waves experience severe instantaneous slamming loads and steep motion responses, as demonstrated by numerical seakeeping models [26]. These severe fluid–structure interactions inevitably transmit to the vehicle chassis and onboard inertial sensors, providing a physical mechanism for the generation of these non-ideal disturbances. These issues limit the generalization of existing hybrid models. Specifically, the algorithm may fail to converge when decoupling sensor noise, broadband disturbances, and high-frequency rotation signals in real time.

#### 3.1.1. Filtering Methods

Traditional filtering methods primarily rely on explicit mathematical modeling or time-frequency domain decomposition of noise signals. In time-frequency processing, wavelet denoising is widely used for its multi-resolution properties. To address the limitation that the single-threshold method disrupts the global structure of coefficients, researchers proposed a divide-and-conquer optimization strategy combining redundant and sparse representations [27]. This method utilizes inverse compressive sensing to optimize low-frequency coefficients and introduces lag correction for boundary effects, thereby effectively suppressing static drift. However, its lengthy algorithmic processing chain, such as orthogonal matching pursuit (OMP) iterative solving, is highly sensitive to prior parameter settings, easily causing severe computational delays and distortion when USVs face high-frequency dynamic impacts.

In the field of state estimation, KF and its variants constitute the foundation for handling random errors. Traditional filtering based on autoregressive moving average (ARMA) models typically relies on stationarity assumptions and extensive offline preprocessing. To overcome this limitation, adaptive filtering and phase space reconstruction (PSR) technologies have been introduced. This approach maps random drift to a finite-dimensional phase space and applies adaptive KF (AKF) for online compensation. Consequently, it bypasses the stationarity assumption and reduces preprocessing overhead, ultimately lowering the standard deviation of random drift [28]. Nevertheless, such methods exhibit strong dependence on the selection of single-step prior parameters, such as the embedding dimension and the time delay.

Therefore, when dealing with high-frequency, nonstationary vibrations and complex temperature drift in USVs, the generalization and modeling capabilities of traditional filtering have reached their limits. Consequently, the academic community is increasingly shifting its focus toward machine learning and deep learning technologies based on nonlinear mapping.

#### 3.1.2. Deep Learning Methods

While traditional filtering struggles in high-dynamic environments, deep learning offers an alternative for MEMS gyroscope denoising. Its strength lies in nonlinear representation and temporal feature extraction. Early explorations primarily focused on recurrent neural networks (RNNs) and their variants. To address the high computational overhead of long short-term memory (LSTM) models, researchers introduced the lightweight deep simple recurrent unit (DSRU) into the denoising field. This method reduces the number of network parameters while maintaining temporal modeling capabilities [29]. Furthermore, neural architecture search (NAS) was employed to minimize the empirical bias of manual design. Results suggest that the network topology itself is a critical factor influencing denoising performance [30].

With further research, the limitations of single-time-series models have become increasingly prominent, particularly their inadequacy in capturing local high-frequency mutation features caused by wave slamming or USV hull vibrations. Therefore, advanced network architectures integrating spatiotemporal multidimensional features have become an important research focus. Hybrid architectures combine one-dimensional convolutional neural networks (1D-CNNs) for local spatial feature extraction with LSTM networks. By incorporating soft attention mechanisms to dynamically weight errors, these models effectively capture the spatiotemporal characteristics of noise [31]. Regarding operational adaptability, a systematic comparison of isomorphic and heterogeneous hybrid RNNs shows that the heterogeneous LSTM-gated recurrent unit (LSTM-GRU) model achieves significant global optimality in balancing static and dynamic random-noise suppression [32]. Similarly, under extreme conditions such as GNSS signal interruptions, a combined prediction model based on wavelet pre-denoising and support vector machines (SVMs) has been proven to enhance system robustness [33]. However, purely data-driven deep networks typically face challenges such as high computational complexity, limited physical interpretability, and susceptibility to overfitting.

In recent years, the cutting-edge trend in denoising has been the deep integration of data-driven and model-driven paradigms. Such cascaded or embedded frameworks aim to simultaneously utilize the nonlinear approximation capabilities of neural networks and the real-time recursive advantages of KF. For instance, embedding a dynamic RNN as a generalized nonlinear autoregressive moving average (NARMA) model into the state equation of the unscented KF (UKF) achieves a sound balance between nonlinear modeling and real-time filtering [34]. For scenarios requiring high static accuracy, a deep cascaded framework was developed. This framework integrates convolutional denoising auto-encoders (Conv-DAE) for reconstruction, a multi-layer temporal convolutional network with an attention mechanism (MultiTCN-Attention) for long-sequence modeling, and particle swarm optimization-based KF (PSO-KF). Experimental results show improved noise suppression compared to the traditional ARMA-KF [35]. Recent advancements further progress toward strict mathematical consistency of network architectures: to address the non-differentiable differencing order in traditional autoregressive integrated moving average models, the straight-through estimator (STE) was introduced. This enabled the construction of an adaptive differencing Elman neural network (ADENN), which was integrated into the cubature KF (CKF). This method not only endows the model with a physical time-series structure but also realizes real-time computation, demonstrating outstanding engineering application potential [36].

In summary, the stochastic error denoising of MEMS gyroscopes has undergone a progressive evolution, progressing from mathematical filtering to pure deep learning, and then to the integration of artificial intelligence (AI) and adaptive filtering. However, for existing deep networks to transition to industrial-grade applications, they still face severe challenges. On the one hand, there is a conflict between the massive model parameters and the constraints of small USVs; on the other hand, the generalization performance of purely data-driven models is highly susceptible to degradation when encountering out-of-distribution data or complex temperature gradients. To systematically outline the technological development of the aforementioned denoising methodologies and reveal their applicability boundaries under complex operating conditions, Table 1 categorizes and compares representative studies. This framework encompasses traditional mathematical filtering, deep learning architectures, and cutting-edge hybrid integration paradigms and evaluates the limitations and application scenarios of various mechanisms in addressing the complex constraints of small USVs.

While deep learning and hybrid paradigms have replaced traditional filtering in stochastic error denoising, current methods remain limited by accuracy, computational power, and generalization in small USVs. While complex deep cascaded and hybrid architectures possess high theoretical accuracy, their massive computational load violates the hardware limitations of micro-platforms. Under real marine high-frequency wave slamming and hull flutter, data-driven models that overly rely on stationary priors are highly susceptible to out-of-distribution failures and filtering divergence. Meanwhile, another core research focus of system engineering lies in the suppression of low-frequency deterministic interference. Such interference also presents strongly nonlinear and hysteretic coupling characteristics, among which temperature drift compensation serves as a typical representative.

### 3.2. Temperature Drift Compensation

After effectively suppressing high-frequency dynamic random noise and ensuring the spectral purity of rotation modulation, the low-frequency deterministic error, dominated by temperature drift, constitutes another core factor in determining the long-term baseline stability of SRINS. In real marine environments, USVs experience severe temperature gradients due to solar radiation and cabin heat dissipation. These thermal variations cause the sensor bias and scale factor to exhibit strong time-varying nonlinear hysteresis, violating the constant drift premise required for rotation modulation. The superposition of this unmodulated blind zone error and the time-varying nonlinear temperature drift is the fundamental cause of the system’s long-endurance azimuthal divergence. Furthermore, under severe sea states, multi-degree-of-freedom vibrations further exacerbate the nonlinear coupling between temperature stress and mechanical rotation stress. Temperature drift compensation follows two paths: hardware control and algorithmic compensation. Hardware control isolates thermal disturbances through physical methods, while algorithmic compensation relies on mapping temperature to error.

#### 3.2.1. Hardware Control

In the error suppression systems of MEMS gyroscopes, hardware optimization constitutes the fundamental layer for accuracy. Given small USVs’ requirements, traditional macroscopic external constant-temperature control schemes are no longer applicable, and the academic community is transitioning from external environmental stabilization to multi-level cooperative control. Current hardware compensation strategies have surpassed single-dimensional constant-temperature control, constructing a multi-level compensation architecture ranging from underlying physical mechanisms to top-level modal control, aiming to provide a superior raw signal foundation for the system.

First, at the level of fundamental physical mechanisms, research focuses on mitigating temperature drift generation at the source. To address the drift caused by damping fluctuations, an in situ dynamic regulation mechanism based on the Joule effect was proposed. By actively regulating the energy dissipation of the mechanical structure, the Quality factor ( Q -factor) fluctuation over a wide temperature range is reduced to 150 ppm, thereby directly suppressing the physical thermal noise source [37]. In addition, the thermal expansion mismatch of packaging materials is another cause of hysteresis nonlinearity. To this end, researchers developed a dual-dimensional sensing architecture integrating temperature and stress. By integrating capacitive stress sensors at the chip anchors, changes in the internal residual stress field are captured directly and in real time. Experiments confirm that drift stability improves by nearly a factor of 3 compared with traditional single-dimensional calibration, effectively overcoming the stress hysteresis challenge [38].

Above the physical layer, the circuit sensing layer focuses on canceling residual environmental disturbances by adjusting parameters of the application-specific integrated circuit (ASIC). To address the limitations of heat-conduction delays and coupling errors in external temperature sensors, a virtual temperature sensor based on the drive-loop feedback voltage was implemented in the ASIC design. This technology utilizes an internal lookup table to correct the scale factor and drift in real time, optimizing the drift instability to 1.9°/h [39]. Complementing the aforementioned method is a more cost-effective pure hardware scheme that utilizes a temperature-variable resistor to dynamically adjust the drive reference voltage; this method effectively cancels the temperature drift of the scale factor, achieving a variation rate of only 1.5% over the entire temperature range with a simple circuit structure [40].

Notably, hardware compensation has extended to the highest modal control level, aiming to address the issues of dynamic performance consistency and high-precision requirements. To prevent dual-mass gyroscopes from losing bandwidth at high temperatures, researchers introduced a pole-zero temperature compensation proportional controller. By dynamically configuring closed-loop poles and zeros, this controller stabilizes the bandwidth at approximately 93 Hz across the entire temperature range, ensuring a clear dynamic response [41]. In pursuit of navigation-grade accuracy, a novel mode deflection strategy was proposed. Through circuit configuration, the drive mode is forced to deflect toward the principal axis of damping, thereby physically canceling the influence of the damping azimuth, and ultimately achieving a navigation-grade bias stability of 0.014°/h [42]. To provide a systematic overview of the aforementioned multi-level compensation mechanisms, ranging from the physical underlying layer to modal control, Table 2 summarizes the innovations and limitations of existing hardware strategies.

Through physical isolation, in situ regulation, and circuit feedback, hardware compensation has significantly reduced the raw error magnitude. However, in the harsh marine operating environments of small USVs, severe wave slamming and variable thermal fields generate complex nonlinear coupled interference. Constrained by the physical limits and power constraints of analog circuits, pure hardware optimization struggles to eliminate high-order nonlinear terms and aging drift within a short transient response time. Therefore, after establishing a low-hysteresis signal foundation at the hardware layer, algorithmic compensation should be introduced to process residual complex errors, maximizing system accuracy through hardware and software synergy.

#### 3.2.2. Algorithmic Compensation

Algorithmic compensation addresses physical limitations without increasing USVs’ resource consumption. This approach establishes a mapping model between multi-physics field stress and residual drift. Consequently, it decouples and eliminates high-order nonlinear errors at the data end that are challenging for hardware circuits to process. In the early stages of this technological evolution, traditional algorithmic compensation primarily relied on polynomial regression fitting based on ambient temperature point calibration. However, static mapping often fails to handle abrupt nonlinear changes and temperature hysteresis in MEMS structures. These effects are prominent across wide temperature ranges in the marine environments of USVs. Consequently, artificial neural networks (ANNs) were introduced to characterize the hysteretic relationship between bias and temperature. This approach helps extend the operational duration of the inertial sensors under severe temperature fluctuations [43].

With further research, the limitation that a single temperature input struggles to comprehensively characterize dynamic thermodynamic processes has become increasingly prominent. Consequently, introducing multidimensional temperature field features has become a key factor in enhancing compensation generalization capability. Researchers incorporated the temperature change rate and temperature coupling terms into Elman neural networks (Elman NNs), which possess dynamic memory capabilities. This method overcomes the limitations of single-input models and improves fitting accuracy for time-varying temperature drift [44]. In SRINS, under complex temperature field environments involving the interaction between self-heating and cold starts, research indicates that traditional piecewise compensation tends to generate step errors. Conversely, introducing the temperature gradient as an additional input feature into backpropagation (BP) neural networks can achieve continuous and smooth global temperature compensation [18]. Metaheuristic strategies are often used to address issues such as local optima and sensitivity to initial weights in neural networks. For example, researchers have employed the genetic algorithm (GA) or the black-winged kite algorithm (BKA) to optimize gated recurrent units (GRUs). These methods improve stability in long-sequence temperature drift modeling while reducing network parameters [44,45].

In signal processing architecture design, the traditional sequential processing paradigm, namely denoising followed by temperature compensation, has been proven to be prone to inadvertently losing useful low-frequency vehicle motion signals. To address this limitation of information loss, complex modal parallel processing frameworks emerged. For instance, researchers developed a parallel architecture combining multi-objective particle swarm optimization (MOPSO), variational mode decomposition (VMD), and an Elman network optimized by the Beetle Antennae Search (BAS) [46]. A parallel framework employs improved complete ensemble empirical mode decomposition with adaptive noise (ICEEMDAN) for signal processing. This method is combined with an extreme learning machine (ELM), which is optimized by the non-dominated sorting genetic algorithm II (NSGA-II) [47]. Utilizing sample entropy classification, these parallel strategies achieve cooperative optimization of high-frequency random noise suppression and low-frequency strong nonlinear temperature drift compensation, reducing the drift instability to a small fraction of its original magnitude.

However, purely data-driven AI models face several limitations, regardless of whether they use unidirectional or parallel architectures. These include poor physical interpretability and a high reliance on offline calibration data. Furthermore, the computational overhead often hinders real-time deployment on embedded systems. In recent years, the cutting-edge direction in this field has witnessed a paradigm shift toward physics-driven mechanisms and sensorless internal parameter fusion. For physical mechanism modeling, a geometric nonlinear variational framework was developed to replace basic curve fitting. This framework superposes the thermal displacement field and nominal vibration. Combined with distributed capacitive stress sensors, this method can directly predict the frequency drift and quadrature error caused by thermal stress, marking a crucial step toward genuinely physically interpretable drift compensation [48]. In electrical and system control, scale factor nonlinearity and zero-rate output (ZRO) drift across the temperature range are linked to coupled phase errors. These errors stem from parasitic capacitance and demodulation link issues. Consequently, closed-loop phase adaptive real-time systems and online phase error compensation mechanisms have been developed [49,50]. These methods operate without external temperature sensors and provide error suppression that outperforms pure algorithmic fitting. Furthermore, recent studies have proposed an innovative multi-parameter fusion compensation (MPFC) model that unifies the characterization of the ZRO temperature drift mechanism by directly extracting electromechanical control parameters, such as drive mode resonant frequency, drive voltage, quadrature voltage, and reference voltage. This method eliminates the reliance on external temperature sensors. It has achieved ultra-low-latency real-time compensation on field-programmable gate arrays (FPGAs), effectively reducing the peak-to-peak ZRO value across the entire temperature range [51]. Table 3 categorizes and benchmarks current algorithmic temperature drift compensation strategies. This table outlines the evolution of algorithm design. The logic shifts from data-driven black-box mapping to computationally intensive parallel processing. It concludes with physics-driven and sensorless fusion, which balances physical interpretability and low latency.

Temperature drift compensation in MEMS gyroscopes has transitioned from black-box fitting to multidimensional parallel networks. The current trend focuses on mechanism perception and sensorless multi-parameter fusion. However, in the complex operating domains of USVs, algorithmic compensation still faces scientific challenges. Further simplifying complex physical thermal stress mechanism models and resolving the compensation robustness problem under long-term service aging and extreme dynamic thermal shocks remain key challenges. Therefore, to navigate these bottlenecks, compensation strategy selection can be driven by scenarios. For resource-constrained small USVs, mitigating drift at the source through hardware-level physical regulation combined with real-time algorithms (e.g., the ADENN-integrated CKF) represents a viable and efficient approach. Conversely, when USVs experience wave slamming where purely data-driven models are susceptible to out-of-distribution failures, the MPFC model is recommended as the superior approach due to its ultra-low latency and physical interpretability.

More importantly, the aforementioned denoising and temperature drift compensation only address errors at the sensor signal layer, providing pure inertial data for the SRINS. However, before the system enters normal rotation modulation and navigation calculations, the vehicle’s initial attitude must be established. Traditional static alignment, relying on gravity and Earth rotation, becomes invalid under moving-base conditions, where USVs experience severe wave swaying. Compensated MEMS data is essential for rapid, high-precision initial alignment under strong disturbances. This alignment determines the accuracy of the spatial framework for the single-axis rotation mechanism. This constitutes the core technical challenge explored in depth in Section 4.

## 4. Moving-Base Initial Alignment

Establishing a reliable initial navigation reference in dynamic marine environments is a fundamental prerequisite for USVs to operate autonomously. The conventional moving-base alignment process typically includes coarse alignment for rapid analytical attitude acquisition and fine alignment for accurate optimal state estimation. MEMS sensors equipped on small unmanned surface vehicles (USVs) suffer from high noise, which degrades the estimation accuracy of misalignment angles. This issue is compounded by continuous wave slamming and high-dynamic angular sway in low-cost MEMS-SRINS. Although single-axis RMT can effectively suppress constant drift, it is insufficient at the physical mechanism level to isolate the complex dynamic kinematic interference introduced by severe sway. Therefore, multi-source-aided alignment, integrating external measurement information from systems such as GNSS, Doppler velocity logs (DVLs), or magnetic compasses, has become an effective approach to suppress alignment divergence and accelerate convergence.

### 4.1. Coarse Alignment

Coarse alignment in the moving-base environment of USVs faces a key challenge. It requires rapidly separating high-frequency linear acceleration interference and unknown base angular motion to establish the initial reference frame. Traditional analytical alignment relies on a small-angle assumption. In contrast, optimization-based alignment (OBA) reframes alignment as an attitude determination problem using continuous vector observations (e.g., solving Wahba’s problem for matrix K eigenvectors). This approach handles large angular motions more effectively [52]. Under complex conditions, this attitude determination technology based on continuous vector observations has been shown to enhance the system’s resistance to non-periodic sway and to improve convergence speed [53].

However, the OBA mechanism heavily relies on the integrity of external observation vectors. To suppress disturbances like initial velocity errors, researchers have optimized the construction of observation vectors. One approach involves canceling dynamic errors from the moving base through algebraic operations, such as vector subtraction [54,55]. Early studies typically assumed frequency-domain separability and adopted low-pass filtering, such as high-order finite impulse response (FIR) filters, for pre-isolation [52]. This strategy is effective under calm sea conditions. However, it fails when USVs encounter non-Gaussian impulsive interference, such as wave slamming or occlusion. These conditions lead to GNSS velocity anomaly jumps. Therefore, integrating adaptive robust estimation has become necessary. To this end, researchers utilized the norm invariance of reference vectors and observation vectors to construct novel test statistics. This mechanism decouples outlier detection from the initial attitude. It also uses robust parameter identification to remove time-varying sensor drift, improving OBA calculations for contaminated observations [56].

Furthermore, in addition to algorithmic improvements, for systems equipped with specialized hardware, such as the RMT inertial navigation system (INS), analytical self-alignment strategies based on dual-position switching provide an alternative. This strategy suppresses interference angular rates and accelerations through dynamic error compensation at both physical and analytical levels. It achieves rapid convergence in swaying environments. Furthermore, it mitigates model mismatch risks often found in filtering methods under strong interference [57]. Based on this, an information-reusing mechanism is introduced during the coarse alignment stage. This strategy provides data support for subsequent filtering state backtracking [58]. However, in complex sea states, the aforementioned coarse alignment schemes still have limitations. There remains a fundamental contradiction between the long observation time required to suppress the high noise of MEMS sensors and time-varying kinematic model errors [59] and the real-time initialization requirement of USVs.

### 4.2. Fine Alignment

Coarse alignment provides the initial attitude, but its residual errors often violate the small-angle assumptions required for fine alignment. Beyond the wave-induced disturbances mentioned previously, fine alignment must resolve challenges from large misalignment angles and poor observability. To solve the linearization error divergence problem triggered by large misalignment angles, early unified alignment frameworks attempted to introduce Rodrigues parameters combined with the second-order extended KF (EKF2) for nonlinear approximation [60]. Furthermore, researchers introduced the ensemble particle filter (EnPF) optimized based on Kullback–Leibler distance (KLD), as well as robust nonlinear filtering integrating variational Bayesian (VB) or maximum correntropy theory [61,62]. These high-order methods aim to fit the true posterior probability distribution as accurately as possible in non-Gaussian and strongly nonlinear scenarios [61]. However, for small USVs with strictly limited computational resources, whether involving complex Jacobian matrix derivations or Gaussian kernel bandwidth optimization, these high-order or particle-based nonlinear filtering methods inevitably incur a significant increase in computational complexity, hindering their practical deployment and preventing them from meeting the real-time constraints of embedded edge computing platforms [63]. Evaluations under dynamic sea states show that traditional nonlinear adaptive filtering has strict application boundaries. In complex marine environments with measurement interference, these methods often induce system noise amplification and model degradation [64].

In recent years, research based on the topological characteristics of Lie groups has established a new geometric approach for fine alignment under large misalignment angles. Mapping the alignment mechanism from the traditional Euclidean space to the SO(3) Lie group allows for the explicit separation of state-dependent measurement noise. This approach eliminates high-order coupling terms often ignored in traditional nonlinear filtering, which improves heading accuracy [65]. A further advancement lies in embedding the entire system into the $SE_{2}(3)$ (double direct spatial isometries) affine framework and adopting a left-invariant error model based on the frozen navigation frame. This model mathematically proves the global log-linear property of the error system. This not only enables the system to achieve robust convergence with the ultra-low computational complexity of the standard KF when facing extreme misalignment angles of ±180° , but also fundamentally eliminates the traditional cascaded boundary between coarse alignment and fine alignment at the underlying logic level [66].

The Lie group framework handles nonlinear challenges from large misalignment angles. However, in the dynamic scenarios of USVs, state desensitization occurs during late-stage fine alignment. Weakly observable states, such as the heading angle, cause filter dependence on external observations to decay prematurely. This constraint limits the convergence speed. Therefore, adaptive robustness and closed-loop feedback mechanisms have become key solutions to improve terminal convergence efficiency. For instance, the variance-constraint KF (VCKF) maintains observation weights by introducing a variance lower bound based on relative convergence. This mechanism creates an excitation effect, reducing the fine alignment time of the classical KF under moored swaying conditions [67]. Similarly, for SRINS, a multi-channel forward-backward filtering method combining backtracking navigation and Sage–Husa adaptive R matrix constraints can fully mine the historical data value of the coarse alignment stage, significantly enhancing robustness against complex interference [68].

Notably, regardless of the fine alignment strategy adopted, the final convergence lower bound of the system is not only limited by the algorithmic computational capability but also deeply coupled with the physical configuration of the sensors. Theoretical analysis shows an explicit analytical mapping between the final fine alignment error and the three-dimensional initial attitude of the IMU [69]. Specifically, the azimuth axis ( Z -axis) sensor demonstrates the highest attitude sensitivity. This fundamental theoretical discovery provides a critical benchmark for sensor pose planning and a priori error budgeting in subsequent moving-base alignment. Guided by these theoretical constraints, recent research has shifted towards introducing complementary multi-source external information—such as GNSS or DVL—to further enhance alignment robustness and precision beyond inertial limits.

### 4.3. Multi-Source Aided Alignment

To overcome the aforementioned physical limitations of the single-axis rotation mechanism and issues such as inherently low observability, the integration of multi-source external information has emerged as a critical strategy. This approach is designed to compensate for the lack of measurement information in moving-base environments and meet the rapid alignment requirements of small USVs. Early research primarily focused on leveraging the vehicle’s motion constraints or manual intervention to enhance the system’s observability. For instance, by introducing the vehicle’s angular velocity as a pseudo-measurement in dual-position alignment and constructing a linear observation model in the body frame, the convergence speed of the azimuth misalignment angle can be significantly improved without adding additional hardware [70]. However, the strong dependence of such methods on specific maneuvering trajectories (e.g., heading rotation) limits their universality on vehicles with complex motion characteristics. Subsequent studies extended angular velocity aiding into body-frame filtering architectures. These methods employ robust strategies (such as Huber M-estimation) to address issues of measurement noise correlation and modeling errors under large misalignment angles. This approach enables rapid alignment without a coarse alignment stage, even when the vehicle exhibits random motion characteristics [71]. This evolution from relying on specific maneuvers to adapting to random maneuvering disturbances reflects the deepening development of aided alignment technologies from ideal laboratory scenarios to complex engineering environments.

For small USVs, the velocity reference information provided by GNSS or DVL serves as the physical foundation for overcoming the heading observability limits of SRINS. Studies have shown that the improvement in initial alignment performance depends not only on the performance of filtering algorithms but also on the in-depth exploration of observability mechanisms. Guiding the vehicle to execute specific turning maneuvers through numerical observability analysis can effectively enhance system observability, thereby providing an excellent excitation trajectory for DVL-aided fine alignment [72]. Nevertheless, actual marine operating conditions are often accompanied by multiple challenges, including GNSS signal denial, missing latitude data, and DVL outlier interference. Accordingly, multi-source data fusion technology that integrates multiple sensors such as DVL, visual odometry, and compasses has been widely adopted to correct the navigation divergence errors of USVs during measurement signal outage periods [73]. To handle extreme navigation conditions, a consistency check based on constructing gravity vectors (CGV) is proposed. This method treats DVL outlier detection as a geometric consistency evaluation. Combined with the robust backtracking filtering algorithm, it can realize moving-base alignment without relying on known latitude information [74]. Notably, regarding the nonlinear issues of low-cost MEMS sensors under large initial misalignment angles, the state transformation unscented KF (ST-UKF) reconstructs the velocity error model by replacing unstable specific force terms with gravity terms. When combined with the dual-position rotation scheme, this algorithm can greatly suppress the adverse effects caused by large MEMS gyroscope drift, exhibiting prominent theoretical advantages in breaking the performance limits of low-cost navigation sensors [75].

Multi-source aided alignment technology has evolved from early simple velocity matching to a multidimensional cooperative architecture encompassing nonlinear geometric modeling and trajectory observability optimization. Distributed multi-sensor data fusion and multi-rate adaptive filtering have been used to resolve spatiotemporal asynchrony issues. These strategies ensure the engineering feasibility of aided alignment under complex operating conditions [75,76]. To systematically outline the developmental progression of moving-base initial alignment technologies, Table 4 summarizes the core innovations, limitations, and application scenarios of the aforementioned representative alignment strategies.

Accordingly, selecting an appropriate alignment strategy requires a comprehensive analysis of specific operating environments. Under general moving-base scenarios, OBA aided by multi-source sensors (such as GNSS or DVL) is generally recommended, as it can effectively handle large angular motions and compensate for the physical blind zone of the single-axis mechanism. Conversely, when USVs operate in severe states with initial misalignment angles, traditional nonlinear filtering may risk noise amplification and increased computational burden. In such challenging cases, the Lie-group geometric framework provides a highly competitive alternative, as its mathematical properties help eliminate high-order coupling terms and facilitate robust convergence while maintaining a relatively low computational complexity.

Based on the above technical development context, although the existing Lie group geometric frameworks and multi-source fusion strategies have greatly expanded the observability boundary of alignment systems, their practical deployment on small USVs equipped with MEMS-SRINS still faces critical challenges. Under complex operating conditions, initial alignment needs to be completed rapidly after system startup. During long-endurance navigation, the inherent uncalibrated residual errors of low-cost MEMS sensors inevitably cause the accumulation or divergence of heading errors. Therefore, simply improving the accuracy of initial alignment can hardly meet practical requirements. It is urgent to introduce a dynamic online azimuth calibration mechanism during system operation, so as to realize the technical paradigm upgrade from accurate initial alignment to long-term steady-state error suppression, which serves as the core research focus of Section 5 in this paper.

## 5. Azimuth Gyroscope Drift Online Calibration

Addressing the unmodulated azimuth gyroscope drift is a critical challenge for medium- and low-precision MEMS devices operating in complex environments. Therefore, achieving rapid online calibration of this error remains a limitation in current system-level calibration research.

### 5.1. Observability Analysis

The accurate estimation of azimuth gyroscope drift fundamentally depends on the system’s observability characteristics. Observability degree (OD) analysis indicates that under moored or non-rotating conditions, the azimuth gyroscope drift is almost unobservable [78]. Without specific angular motion modulation, certain inertial sensor errors remain poorly observable [79]. Addressing the time-varying characteristics induced by rotation, the piecewise constant system (PWCS) observability theory and its associated total/stripped observability matrices (TOM/SOM) lay a solid theoretical foundation for the subsequent scientific design of rotation calibration sequences [80].

Based on the above theories, the research focus in this field has gradually shifted from passive observability evaluation to active rotation path design. Global observability theory was introduced into rotation systems, establishing the joint design criteria for rotation axes and dwell positions [81]. Regarding the quantitative evaluation system, the condition number was introduced to diagnose the ill-conditioning of the observation equations, thereby improving the numerical stability of drift estimation by optimizing rotation angles, and Covariance-based OD was used to optimize a 30-position rotation scheme [82]. Recent system-level calculations show that continuous rotation paths outperform traditional piecewise indexing strategies for full-state excitation [83,84]. Additionally, the dynamic online calculation of OD has been deeply integrated into the algorithmic layer, for example, serving as an information-sharing factor to drive adaptive federated filtering, thereby flexibly addressing complex time-varying environments [85].

Although multi-axis and multi-position observability schemes possess a sound theoretical framework, they rely heavily on high-precision turntable equipment and require lengthy calibration durations (typically more than one hour). Such methods generally need to be implemented under static conditions, which conflicts with the rapid calibration requirements of small unmanned surface vehicles (USVs). High sea state operating conditions fail to satisfy static assumptions and simultaneously aggravate the nonlinear coupling effect of MEMS sensor errors.

### 5.2. Algorithmic Calibration

To mitigate the nonlinear coupling risks induced by the aforementioned mechanical calibration in high sea states, researchers are developing more efficient algorithm-level calibration strategies under limited maneuvering constraints.

Within the KF framework, introducing additional measurements is an effective approach to enhance calibration accuracy. For instance, under stationary single-axis rotation conditions, introducing horizontal attitude and heading measurements significantly enhances the observability of the Z -axis drift [86]. Building on this, combining physical rotation modulation with specific dead reckoning algorithms further verifies the feasibility of automatically suppressing residual sensor errors in harsh GNSS-denied environments [87]. To handle dynamic interference during mooring, researchers use an adaptive robust KF (ARKF). This approach combines the VB method, multi-factor robust optimization, and angular rate extended measurements to prevent filter degradation caused by model errors [88]. To address nonlinear error accumulation from frequent turning or severe maneuvers, a sliding-window-based vector observation strategy is used. This approach improves in-motion calibration stability by truncating historical accumulated errors [89]. Furthermore, integrating Huber M-estimation with the robust filtering framework enables real-time outlier rejection from external auxiliary sensors such as DVLs, thereby ensuring the accuracy of calibration parameter identification in complex marine environments [90]. To improve fault tolerance in nonlinear environments, multivariate cumulative sum control charts are combined with the CKF. This approach increases detection sensitivity to slowly varying drift and enhances the robustness of high-precision navigation systems [91].

To improve multi-parameter calibration efficiency and environmental adaptability, an eight-step continuous rotation scheme combined with KF enables the excitation and fitting of multiple parameters within 10 min under non-constant temperature conditions [92]. Small vehicles often lack precision turntables during field missions. To address this, a self-calibration method for micro IMUs adopts only tilted installation and gravity references. This approach extracts system error parameters through static and rotation tests [93]. Meanwhile, manual rapid field calibration based on zero-velocity update technology significantly reduces algorithmic dependence on complex testing environments, enabling efficient calibration of low-cost MEMS sensors [94].

However, KF-based calibration methods consistently face limitations, including nonlinear coupling between attitude and IMU errors, small-angle assumptions, and a severe reliance on noise priors. To resolve this issue, optimization-based algorithms have become key solutions in recent years. By introducing the environment function matrix (CFM) theory, complex error propagation integral terms are transformed into a linear least-squares observation matrix, thereby eliminating the reliance on KF parameter tuning [95]. Furthermore, the introduction of bilinear embedding and adjoint transition matrix theory mathematically eliminates the nonlinear coupling terms between attitude and IMU errors, enabling stable convergence even with large initial attitude errors up to 60° [96]. Although optimization methods exhibit excellent robustness, they typically require buffering a massive amount of historical integral data and relying on complex multi-position rotation trajectories. The need for high computational power and complex maneuvering conflicts with USV onboard hardware. Typical MEMS setups lack sufficient resources and are restricted to single-axis rotation.

### 5.3. Mechanical Error Identification

In addition to the aforementioned algorithmic calibration targeting inherent sensor errors, the physical introduction of the single-axis rotation mechanism inevitably introduces mechanical defects of its own. From the perspective of error mechanism analysis, such system errors caused by non-ideal physical integration are primarily categorized into two types: static installation misalignments, such as the non-orthogonal angle between the IMU and the rotation axis, and dynamic rotation-induced errors, such as shaft sway and unidirectional electromagnetic or frictional coupling. If these errors are not effectively decoupled, they will severely degrade the calibration accuracy of the azimuth gyroscope drift.

Targeting static installation misalignments, particularly the challenging non-orthogonal conditions in multi-axis or gimbaled systems, the academic community has proposed various isolation mechanisms. For low-cost MEMS chips, a self-calibration method requiring only an ordinary inclined plane and a gravity reference effectively identifies the shaft misalignment angle [93]. For space-constrained engineering applications, a practical installation-error calibration algorithm was proposed to quantitatively compensate for the deviation between the instrument axis and the vehicle structural axis in single-axis systems [87]. To address more complex error coupling, the periodic averaging method is utilized to separate the shaft sway error from the PID control error, constructing a quadratic temperature model for the non-orthogonal angle [97]. The consistency of attitude during stationary periods is used as a physical invariant in the observation equation. This approach enables the simultaneous convergence of non-orthogonal angles through a single rotation sequence [98].

Regarding dynamic rotation-induced errors, unidirectional rotation coupling errors are revealed, and adopting a reciprocating rotation strategy is recommended for physical suppression [99]. Further analysis of the heading effect in initial alignment reveals that its primary driver is the residual drift, which can be precisely compensated online through four-position analytical calibration [100]. To further isolate mechanical disturbances at the physical level, photoelectric encoders are introduced into the rotation architecture to construct a relative attitude observation model. This hardware-assisted method effectively separates random errors arising from the mechanism’s non-ideal transmission characteristics, significantly improving the calibration accuracy of gyroscope parameters [101].

However, it must be noted that the two types of structural error identification methods primarily fall under preprocessing or semi-offline work to ensure attitude accuracy. On the low-cost MEMS-SRINS hardware mounted on small USVs, constrained by machining accuracy, the aforementioned mechanical defects are more significant. Additionally, the specific gimbal structure inherently limits the excitation of error terms, leaving some parameters in a weakly observable state [79]. To comprehensively present the current research status discussed in this section, Table 5 summarizes the core methodologies, innovative contributions, and inherent limitations of existing azimuth gyroscope drift calibration strategies.

In summary, although global observability design and high-dimensional nonlinear optimization algorithms have achieved significant breakthroughs in theoretical completeness, their strict reliance on multi-axis full-space maneuvering or massive historical data caching makes them difficult to deploy on micro USVs equipped with single-axis rotation architectures. Furthermore, existing mechanical error separation primarily remains at the static preprocessing stage, struggling to achieve real-time dynamic decoupling amid severe wave swaying. Therefore, the current field of moving-base online calibration urgently needs to eliminate reliance on high-dimensional maneuvering and to construct a lightweight azimuth gyroscope drift estimation model under the simplified architecture of pure single-axis continuous rotation, balancing strong dynamic disturbance rejection with low computational overhead. Implementing an effective online calibration strategy dictates a balance between onboard computational capacity and available field facilities. For field missions without precision turntables, inclined plane methods offer a viable solution. These methods effectively reduce the dependence on complex testing facilities. To overcome nonlinear coupling and the reliance on noise priors in traditional filtering, optimization-based algorithms such as the CFM offer a theoretically robust alternative. In resource-constrained scenarios, lightweight KF-based strategies combined with continuous rotation schemes provide a practical balance. This approach facilitates multi-parameter fitting within a brief timeframe. Importantly, it avoids the massive historical data caching typically required by complex optimization methods.

More importantly, regardless of how thorough the underlying denoising, temperature drift compensation, and azimuth calibration of the SRINS are, pure inertial navigation remains, at its core, a relative dead-reckoning system, and its position and velocity errors will inevitably exhibit global drift over long-endurance operations. To obtain absolute navigation position and attitude with bounded errors, the system must ultimately rely on deep integration with external GNSS signals. However, in harsh marine operating environments, GNSS is highly susceptible to wave occlusion, multipath effects, and electromagnetic spoofing, exhibiting strong non-Gaussian interference or even long-term denial-of-service. This review also focuses on adaptive robust integration architectures compatible with single-axis rotation. This design is specifically tailored for extreme conditions where external observations are severely degraded. This is also the primary challenge to be addressed in Section 6, Robust GNSS/SINS Integration.

## 6. Adaptive Robust GNSS/SINS Integration Method

After completing the error decoupling and initial alignment based on single-axis rotation, the system formally enters the dynamic navigation and positioning stage. Currently, SINS and GNSS are the two most widely applied positioning methods. Although RMT improves dead reckoning accuracy for low-cost MEMS, the error accumulation of SINS persists. This characteristic prevents the independent execution of long-endurance missions. While GNSS can provide high-precision absolute position and velocity over long periods, it has a low data update frequency and poor anti-interference capability, making it highly susceptible to failure in complex operating environments. Therefore, constructing a GNSS/SINS integrated navigation system to complement their respective advantages has become an effective choice.

Currently, under complex marine operating conditions, the low-cost integrated systems of small USVs face severe internal and external challenges. In harsh seas, vehicles perform large maneuvers like sharp turns, rapid speed changes, and wave piercing. These actions cause significant errors in the dynamic models of traditional filters. GNSS signals are vulnerable to wave occlusion, spray, multipath effects, and electromagnetic interference. These factors cause non-Gaussian outliers or transient loss of lock. Integrated navigation systems are highly sensitive to high-frequency maneuvers and erratic GNSS signals. To prevent filter divergence, researchers should develop more adaptable and robust integration mechanisms.

### 6.1. Adaptive Filtering

To address dynamic model mismatch and time-varying noise caused by high-frequency maneuvering of small USVs and complex sea states, adaptive filtering is the fundamental mechanism for enhancing the resilience of the navigation system. In dynamic, variable real-world engineering environments, the statistical characteristics of measurement noise are often unknown or exhibit time-varying features only slowly. When a priori statistical assumptions deviate from actual operating conditions, the traditional KF is highly susceptible to degraded accuracy or even divergence. Addressing the issue of unknown noise covariance, early studies introduced an improved Sage–Husa noise statistic estimator that compensates for the lack of a priori knowledge under complex marine operating conditions [102]. However, such methods are prone to suffering from suboptimal convergence. In contrast, VB inference provides a rigorous online estimation framework. Still, traditional VB algorithms involve massive iterative computational overhead in high-dimensional systems, making it difficult to meet the computational constraints of low-power embedded platforms on USVs. An equivalent variational iteration formula for measurement noise covariance was developed for highly dynamic alignment. This approach reduces computational complexity while maintaining adaptive effectiveness, improving real-time engineering feasibility [63].

On the other hand, when the USV system encounters a process-model mismatch due to severe maneuvers, a single kinematic model is often insufficient to capture the complex dynamic changes. To facilitate a smooth transition between models, the interacting multiple model (IMM) method is widely adopted. By integrating kinematic and dynamic models into the IMM framework, the system can effectively adapt to abrupt changes in vehicle velocity and sideslip conditions [103]. To substantially extend this multi-model advantage to the marine domain, the hybrid IMM (HIMM) architecture was proposed to address the time-varying nature of complex hydrological characteristics [104]. Furthermore, to address the inherent limitation of traditional IMM, which relies heavily on fixed Markov prior transition probabilities, the recursive maximum likelihood method was introduced to dynamically update the transition probability matrix [105]. The further development of an adaptive state-transition mechanism significantly improves the multi-model system’s sensitivity to unknown ocean currents and dynamic disturbances [106].

In recent years, adaptive mechanisms have shown a trend of deep coupling of multiple strategies, aiming to cooperatively suppress process errors and measurement errors within a unified framework. For example, integrating the VB algorithm as an adaptive sub-model of the IMM achieves parallel processing of time-varying noise estimation and dynamic model switching [107]. Researchers applied the IMM method to fuse standard and adaptive CKF in parallel. The integration incorporates fading and strong tracking factors based on the Mahalanobis distance for improved performance in tightly coupled systems [108]. The test hypothesis enables the system to switch dynamically between VB-based adaptive mode and a generalized mutual information-based hybrid robust mode. This dual-mode approach resolves the limitations of using a single mechanism [109].

Adaptive filtering effectively mitigates the impact of time-varying noise variance and model mismatch within the Gaussian framework. However, facing more severe non-Gaussian multipath abrupt changes and outlier interference under extreme sea states, mechanisms that rely solely on parameter estimation often fail due to convergence lag. Therefore, introducing robust estimation has become the fundamental approach to reconstructing the cost function.

### 6.2. Robust Estimation

When the system faces severe GNSS multipath, abrupt changes, or intermittent signal occlusion caused by large waves under harsh sea states, errors often exhibit strong, heavy-tailed, non-Gaussian characteristics. In complex multipath and non-line-of-sight environments, signal reflection and occlusion violate the fundamental assumptions of standard filtering. These conditions lead to rapid degradation in navigation performance [110]. Facing such adverse observation conditions that severely violate the Gaussian assumption, robust estimation represented by M-estimation and information-theoretic learning (ITL) demonstrates strong capabilities in actively isolating abnormal outliers.

Traditional robust filtering primarily focuses on the measurement end. However, under strong maneuvering conditions, such as small USVs encountering wave impacts, the state transition process also generates non-Gaussian uncertainties. State estimation models grounded in USV physical dynamics have explicitly verified that these severe marine disturbances inherently manifest as heavy-tailed process noises with unknown statistical parameters [111]. To this end, Huber M-estimation is introduced into the cascaded iteration to adjust the a priori covariance, effectively suppressing process uncertainties caused by severe maneuvers [112]. Both predicted states and measurements often suffer from non-Gaussian noise. To resolve this, the maximum correntropy criterion (MCC)-based generalized interacting multiple model KF (GIMM-KF) suppresses outliers in both the dynamic model and the observations [113]. To address the more severe heavy-tailed noise contamination in surface environments, Student’s *t*-distribution with different degrees of freedom is used to model process and measurement noise, respectively. By integrating VB inference to approximate the posterior as a Gaussian distribution, this method achieves strong robustness against non-Gaussian noise while maintaining closed-form recursion [114]. Similarly, an improved statistical similarity measure KF (ISSMKF) with adaptive degrees of freedom has also been proven to reject measurement outliers in extreme waters effectively [112].

Regarding robust criteria based on information theory, the MCC and minimum error entropy criterion (MEE) are widely utilized for their ability to effectively capture high-order error statistical characteristics. For instance, the combination of adaptive MCC and innovation covariance matching technology effectively mitigates severe measurement errors and Gaussian mixture noise in complex marine environments [115]. To handle time-varying noise and non-Gaussian multipath in deep-water environments, VB inference is integrated with MCC. This approach suppresses measurement and model anomalies by adaptively adjusting the kernel bandwidth [116]. Furthermore, to counteract the more extreme, strong, non-Gaussian noise in shallow-water environments, the fiducial point-based adaptive minimum error entropy (AMEEF) criterion ensures that the system maintains filtering consistency even under harsh conditions with injected outliers [117]. Despite these advantages, the robust estimation framework inherently introduces highly nonlinear cost functions. Therefore, for resource-constrained USV platforms, optimizing the hyperparameter tuning and iterative overhead of these information-theoretic methods remains essential for practical large-scale deployment.

### 6.3. Multi-Source Fusion Architectures

Although algorithmic optimization enhances anti-interference capabilities, under the strict constraints of limited computational power and power consumption, relying solely on algorithmic-level compensation struggles to overcome physical limits. The evolution of fusion architectures is the critical factor determining the system’s survival limit in denied environments. Early engineering applications primarily adopted loosely coupled architectures using position or velocity as measurement inputs [118]. However, once transient denial or long-term interruption of GNSS signals occurs, measurement updates in loose coupling will fail, causing errors to diverge rapidly.

To overcome this vulnerability, cutting-edge fusion architectures introduce data-driven generative mechanisms. For example, utilizing LSTM for high-fidelity prediction and self-learning of navigation states or external measurement outputs has been proven to effectively maintain the continuity of the filtering update link during external signal denial [119,120]. Furthermore, measurement difference enhancement and dynamic adjustment of forgetting factors combined with the adaptive incremental KF (AIKF) can effectively maintain the identification accuracy of MEMS errors during severely limited measurements or maneuvering interference [121]. Although combining AI methods such as complementary ensemble empirical mode decomposition (CEEMD) and CNN-LSTM demonstrates remarkable efficacy in maintaining short-term accuracy, their essence lies in data-driven black-box fitting, without increasing the system’s observability from the physical equation level [122]. Therefore, USV systems are prone to divergence during long-term denial that exceeds the generalization limits of the training data.

Therefore, tightly coupled architectures using raw sensor measurements, combined with white-box methods that utilize physical mechanisms and geometric manifolds, represent a growing trend for resolving these challenges:

- Marine Multi-Source Augmentation: The core advantage of tight coupling lies in maintaining system observability even when the number of available observation satellites is insufficient for independent positioning. Constructing unified observation equations with DVL beam frequency shifts and USBL raw ranges ensures stable updates. This approach maintains system performance even when acoustic beams are limited [123]. A dual-transponder slant range differential architecture, aided by inertial navigation time backtracking, is proposed. This design cancels common array errors at the physical mechanism level [124]. However, this deep decoupling relies heavily on the strict deployment of external beacons, increasing the engineering costs of marine exploration.
- Error Lie Groups and Geometric Manifolds: The traditional linearized KF fails to handle the large misalignment angles and covariance distortions induced by severe sea states. This limitation is particularly evident during intense wave swaying of USVs. The iterated equivariant filter (I-EqF) is introduced into GNSS/SINS tight integration, leveraging the symmetry of Lie groups to decouple the error evolution from the true trajectories, significantly improving global consistency under large initial misalignment angles [125]. However, this improvement in manifold consistency comes at the cost of a high model derivation threshold and the computational overhead of computing the Jacobian matrix.
- Heterogeneous Sensing and GNSS-Denied Navigation: Targeting complex multi-sensor scheduling, a parallel architecture with decoupled measurement updates (interactive sensor-independent-update, ISIU) achieves soft cascading and fault-tolerant access of sensors [126]. Regarding the fault-tolerant discrimination mechanism, the combination of robust estimation and sliding window testing improves the detection capability of the tightly coupled system for small faults [127]. Additionally, the adaptive IIR/FIR fusion filtering architecture effectively enhances the system’s resistance to model uncertainty and transient noise by dynamically adjusting mixing probabilities based on the residual covariance [128]. Meanwhile, the GIMM framework uses Gaussian mixture models to capture nonstationary characteristics, enabling dual adaptive switching between motion and noise modes [113]. In GNSS-denied scenarios, a tightly coupled architecture integrates a single-axis rotation MEMS-INS with odometer increments. This design uses RMT to reconstruct heading angle observability, ensuring navigation for miniaturized platforms [7].

Multi-source fusion architectures are undergoing a paradigm shift from data-driven loosely coupled fitting to tightly coupled reconstruction, deeply integrating physical mechanisms and geometric manifolds. In the future, the core challenge of next-generation robust navigation architecture design lies in overcoming the computational burden of high-dimensional nonlinear state equations while ensuring the flexibility of heterogeneous sensors. Clearly, the pursuit of absolute system robustness often comes at a high cost. Although traditional adaptive and robust filtering mechanisms (such as IMM, VB, and MCC) can keep computational costs within a controllable range, they face physical limits in maintaining state observability under extreme denied environments. Manifold-based and physics-enhanced architectures provide high survivability during long-term GNSS denial. However, these methods require complex nonlinear derivations or additional hardware. To comprehensively present the evolutionary history of robust GNSS/SINS integration, the key methodologies, innovations, and inherent limitations discussed above are systematically synthesized in Table 6.

Systematically reviewing the aforementioned multidimensional technological evolution provides a comprehensive comparison of current mainstream robust GNSS/SINS integrated navigation strategies. Research demonstrates that although these cutting-edge methods offer significant advantages for addressing specific interferences, deploying them on small USVs equipped with single-axis rotation architectures generally poses an engineering dilemma: trading computational power for accuracy. High-precision robust estimation, such as VB and ITL, often exceeds the real-time computational limits of low-power chips due to repeated iterations. Similarly, geometric manifold architectures require complex derivations, while AI data-driven predictions lack physical observability. Under extreme sea states, these approaches risk system divergence or exceeding hardware constraints. Therefore, a promising direction for next-generation robust fusion architectures of small USVs is to explore the synergy between physical-layer modulation and lightweight algorithms. Future research should focus on embedding the deterministic physical period of single-axis continuous rotation as a constraint into robust filtering. This simplified architecture utilizes physical constraints to reduce the algorithmic search space. The result is a balance of low computational cost, high robustness, and global observability. Thus, a suitable integration architecture requires a comprehensive evaluation of environmental harshness and computational constraints. Adaptive filtering performs well in conventional dynamic environments, suppressing time-varying noise and model mismatch in Gaussian systems. Nevertheless, harsh sea conditions induce heavy-tailed non-Gaussian interference and multipath effects, where robust estimation criteria such as MCC can effectively eliminate outliers. Long-term GNSS denial threatens loosely coupled systems, whereas tightly coupled architectures exhibit greater robustness.

## 7. Discussion

This review systematically summarizes the development and evolution of SRINS and its integrated navigation technologies in complex marine environments. Although existing architectures have made significant progress in error decoupling and robust fusion, they still face critical physical and algorithmic barriers in practical engineering deployment and in extreme environmental conditions. Before delving into specific challenges, it is essential to retrospectively evaluate the transferability of the methodologies reviewed in the previous sections. To systematically address the navigation limitations of USVs, this article incorporates advanced algorithms originally developed for adjacent domains, including land vehicles, autonomous underwater vehicles, and mobile robots. The transferability of these technologies to USVs can be strictly categorized as either direct or analogical. Direct transferability primarily applies to the underlying mathematical architectures, such as the basic topologies of deep neural networks, Lie group geometry frameworks, and the fundamental physical error models of MEMS sensors. However, the practical deployment of these algorithms is strictly analogical. Unlike land vehicles with wheel odometry or autonomous underwater vehicles in stable deep-water fields, small USVs lack absolute static moments and face complex marine environments. Consequently, external measurement updates (e.g., replacing zero-velocity updates with DVLs) and kinematic constraints cannot be applied out of the box. They require analogical adaptation and retraining to accurately capture the complex structural interactions unique to surface vessels.

### 7.1. Current Challenges

#### 7.1.1. Hardware Limitations

The error accumulation of low-cost MEMS inertial sensors in extreme dynamic or long-endurance missions remains the primary physical constraint on the long-term autonomy of micro USVs. Under real and complex marine operating conditions, traditional stationary noise assumptions, such as Allan variance analysis, can no longer accurately capture the nonstationary noise characteristics of the rapid drift of low-cost IMUs [130]. To mitigate pure inertial divergence, introducing vision or artificial landmarks to provide external constraints has become a mainstream low-cost alternative [131]. However, this severe reliance on a priori external environments not only imposes strict operational deployment limitations but also introduces vulnerability to sudden changes in illumination and line-of-sight occlusion. More critically, in surface environments with severe swaying or extreme texture scarcity, visual sensors are highly susceptible to severe feature instability and degradation in extraction, putting the hardware perception layer at risk of direct failure [132,133].

#### 7.1.2. Computational Burden

The substantial leap in the performance of high-precision fusion algorithms often comes at the expense of computational resources and real-time performance. In recent years, although emerging optimization-based methods demonstrate absolute advantages in global consistency, their engineering complexity and strict parameter tuning requirements, such as sliding window size and lag marginalization length, result in a computational load significantly higher than that of the traditional EKF [134,135]. High-frequency, tightly coupled tasks and complex integer ambiguity resolutions require continuous marginalization operations. This process demands too much computational power for typical embedded platforms [136]. Similarly, in visual-inertial navigation systems, the joint nonlinear optimization for maintaining binocular point-line features, as well as processing complex multi-code calibration solutions under strict time synchronization, significantly exacerbates the computational burden of micro USVs [133,137]. This has become a core obstacle to the transition of cutting-edge algorithms from the laboratory to low-cost, large-scale commercial applications.

### 7.2. Future Trends

#### 7.2.1. Factor Graph Optimization

Constrained by the errors of traditional filtering, multi-sensor fusion architectures are undergoing a paradigm shift toward factor graph optimization (FGO). Although FGO requires high computational power, its flexibility and mathematical rigor make it suitable for addressing frequent occlusions and complex environments [138]. The FGO architecture incorporates precise IMU pre-integration models, including Earth rotation effects. Compared to traditional models, this approach improves attitude estimation and provides unified spatial alignment for heterogeneous sensors [134,135]. For anti-interference, FGO enables refined robust kernel functions and dynamic weighting. This architecture evaluates GNSS pseudorange quality via Mahalanobis distance to adjust satellite weights. Furthermore, integrating ambiguity propagation into real-time kinematic or INS tight coupling maintains continuous navigation constraints [136,139]. Future FGO research targeting USV-SRINS shows potential to expand in two dimensions: first, embedding the single-axis rotation kinematic period as an a priori constraint edge into the graph; second, exploring graph sparsification and adaptive sliding window strategies to balance high accuracy with the limited computational power of small USVs.

#### 7.2.2. Deep Learning Architectures

Addressing the nonlinear noise and complex temperature drift that mechanical mechanism models struggle to characterize accurately, deep learning is becoming a key force in reshaping the underlying logic of navigation. Early methods typically used two-stage architectures, combining deep learning front-ends for feature extraction with traditional filtering back-ends. For example, these systems perceive environmental abnormalities to adaptively adjust filtering weights [132]. Researchers are increasingly adopting data-driven approaches. For example, CNNs and bidirectional LSTM networks can extract complex noise patterns from raw inertial data [130]. Alternatively, embedding vehicle-specific kinematic constraints into the loss function improves short-term pure inertial navigation [140]. However, the black-box characteristics of end-to-end models limit their interpretability and generalization. Consequently, these models may fail when facing marine impacts outside their training distribution. Future work should establish standardized benchmarks to balance network topology, window length, and real-time performance [141]. Ultimately, the field should shift toward physics-informed neural networks that incorporate established dynamic equations.

#### 7.2.3. GNSS-Denied Navigation

In GNSS-denied scenarios, such as severe interference or signal blockage, the resilience of USV navigation systems is critical. Future navigation systems will move beyond single backup sensors toward environmental semantic mining and on-demand switching of heterogeneous data. For instance, when USVs navigate in restricted under-bridge waters, actively adjusting the visual perspective and extracting stable structural features of bridge piers can help avoid the severe interference caused by dynamic water-surface ripples and reflections [133]. Meanwhile, in safety-critical tasks such as high-precision automatic berthing, tightly coupled technology based on multi-view marker arrays and the error-state KF can achieve seamless, smooth switching and cross-validation between GNSS and vision [131,137]. This strategy of utilizing local environmental semantic priors to cancel global error drift will become the effective path for small USVs to achieve highly reliable positioning in denied waters.

In summary, low-cost integrated navigation is transitioning from single-mechanism deterministic modeling to probabilistic inference driven by data, physics, and optimization. Future breakthroughs require overcoming the computational and generalization limits of high-order FGO and deep learning. This enables a lightweight, resilient fusion architecture featuring computational adaptability, scene perception, and error self-healing for resource-constrained small USVs.

## 8. Conclusions

Small USVs require reliable navigation in complex marine environments. The MEMS-SRINS provides a competitive solution. Physical rotation modulates sensor errors perpendicular to the rotation axis. However, mechanical modulation alone cannot resolve the dynamic coupling, unmodulated azimuth drift, and non-Gaussian disturbances caused by complex sea states. Consequently, current research is shifting from hardware modulation toward algorithmic compensation and multi-source fusion.

This article systematically evaluates four key technologies for the deployment of MEMS-SRINS. First, sensor error compensation strategies are evolving from traditional mathematical filtering to physics-driven fusion and hybrid deep learning. Second, moving-base initial alignment utilizes Lie group frameworks and multi-source information to mitigate large misalignments and wave sway. Third, azimuth gyroscope calibration has transitioned from passive observability analysis to active, lightweight online methods. Finally, robust GNSS/SINS integration employs tightly coupled architectures and robust estimation to improve resilience against multipath interference and GNSS outages.

Despite these algorithmic advancements, an engineering conflict remains. High-accuracy precision models require significant computational resources, which small USVs often lack. Therefore, future performance improvements will rely on architectural innovations rather than merely increasing algorithmic complexity. For example, embedding the periodic features of single-axis rotation as constraints into factor graph optimization and adopting physics-informed deep learning can help balance computational cost, system robustness, and observability. This synergy of physical mechanisms and data-driven methods provides a practical paradigm for the long-endurance autonomy of marine robotics.

## Figures and Tables

**Table 1:** Stochastic error denoising strategies.

Category	Method	Innovation	Limitations	Application Scenarios
Traditional Filtering	Inverse compressed sensing combined with lag correction [27]	Divide-and-conquer optimization combining redundant and sparse representations; Low-frequency coefficient optimization for static drift suppression	Complex algorithmic chain involving OMP iterative solutions; High sensitivity to prior parameter settings; Unverified adaptability under high-dynamic maneuvering	Static drift suppression in controlled environments
	PSR integrated with AKF [28]	Random drift mapping to finite-dimensional phase space; Bypassing strict stationarity assumptions; Reduction of offline preprocessing overhead	Strong dependence on single-step prior parameters; Limited generalization under variable temperatures and complex dynamic conditions	Online iterative compensation for non-stationary signals
Deep Learning Architectures	Lightweight DSRU [29]	Significant reduction of network parameters via lightweight architecture; Retention of strong temporal modeling capabilities	Inadequacy in capturing local high-frequency transient features	Edge devices requiring low computational overhead
	NAS optimizing RNN [30]	Automated topology search mitigating manual network design bias; Identification of structures maximizing denoising performance	High training complexity associated with automated search; Potential black-box interpretability issues	Automated optimization of network topology
	1D-CNNs integrated with LSTM and soft attention mechanisms [31]	Extraction of local spatial features via convolutional layers; Dynamic error weighting via soft attention mechanisms handling temporal dependencies	High computational complexity induced by multi-module integration	Complex noise environments requiring spatiotemporal feature extraction
	Heterogeneous LSTM-GRU model [32]	Global optimality in balancing static and dynamic random noise suppression	Purely data-driven nature lacking physical interpretability	Scenarios requiring balanced performance in static and dynamic phases
	Wavelet pre-denoising combined with SVM [33]	System robustness enhancement against external signal interruptions via combined prediction models	Susceptibility to overfitting; Limited real-time recursive capability	Navigation reliability maintenance during GNSS signal outages
Hybrid Paradigms	Dynamic RNN embedded as a NARMA model into UKF [34]	Equilibrium between nonlinear modeling and real-time filtering via state equation embedding	Increased computational load induced by embedded neural calculations	Real-time filtering requiring nonlinear approximation
	Cascaded framework integrating Conv-DAE, MultiTCN-Attention, and PSO-KF [35]	Deep cascaded structure for comprehensive signal reconstruction and long-sequence modeling; Significant noise suppression improvement over traditional models	High architectural complexity and massive computational cost	High-precision static applications
	ADENN utilizing STE integrated into CKF [36]	Resolution of non-differentiable differencing orders; Strict mathematical consistency with physical time-series structures; Real-time ultra-low latency computation	Unverified performance under extreme temperature gradients and out-of-distribution impacts	Real-time engineering implementation in high-speed embedded systems

**Table 2:** Hardware-level temperature drift compensation strategies.

Category	Method	Innovation	Limitations	Application Scenarios
Physical Mechanism Layer	In situ dynamic regulation utilizing the Joule effect [37]	Active adjustment of energy dissipation compressing Q-factor fluctuations to 150 ppm; Direct suppression of physical thermal noise sources	Requirement for active energy input; Restriction by physical limits of analog circuits	Mitigation of drift induced by damping fluctuations at the source
	Dual-dimensional temperature and stress sensing [38]	Integration of capacitive stress sensors at chip anchors capturing residual stress fields; Near threefold improvement in drift stability	Increased fabrication and packaging complexity accommodating stress sensors	Resolution of hysteresis nonlinearity caused by thermal expansion mismatch of packaging
Circuit Sensing Layer	Virtual temperature sensor utilizing ASIC feedback [39]	Modification of scale factor and drift utilizing drive loop feedback voltage and lookup tables; Elimination of thermal conduction delay, achieving 1.9°/h drift instability	Dependence on accurate lookup table modeling; Limited capability against high-order nonlinear terms	ASIC designs requiring the elimination of external sensor coupling errors
	Dynamic drive reference voltage adjustment [40]	Cancellation of scale factor drift to 1.5% variation utilizing temperature-variable resistors; Simple and cost-effective pure hardware structure	Lack of flexibility for complex error modeling; Susceptibility to analog process variations	Low-cost hardware implementations prioritizing scale factor stability
Modal Control Layer	Pole-zero temperature compensation [41]	Dynamic configuration of closed-loop poles and zeros; Stabilization of bandwidth near 93 Hz across full temperature range	Controller design complexity; Strict bounding by analog circuit bandwidth limits	Dual-mass gyroscopes suffering from bandwidth narrowing at high temperatures
	Mode deflection strategy [42]	Forcing of drive mode deflection to the damping principal axis; Physical nullification of damping azimuth influence, achieving 0.014°/h stability	High implementation difficulty; Inability to address long-term aging drift	Navigation-grade systems requiring superior raw signal foundations

**Table 3:** Algorithmic temperature drift compensation strategies.

Category	Method	Innovation	Limitations	Application Scenarios
Neural Networks and Optimization	ANN utilizing hidden layer mapping [43]	Characterization of hysteretic nonlinear relationships via nonlinear fitting; Superiority over static polynomial regression	Inherent black-box nature lacking physical interpretability; High reliance on offline calibration data	Extension of AHRS operational duration under severe temperature changes
	Elman NN incorporating multidimensional inputs [44]	Incorporation of temperature change rates and coupling terms; Improvement of time-varying drift precision via dynamic memory capabilities	Susceptibility to local optima; Sensitivity to initial weight settings	Dynamic environments requiring precise characterization of time-varying thermal drift
	BP NN utilizing temperature gradient inputs [18]	Utilization of internal and external temperature differences as input features; Achievement of continuous global compensation avoiding step errors	High training complexity; Requirement for accurate gradient feature extraction	Complex thermal fields involving interaction between self-heating and cold starts
	GRU optimized by BKA [45]	Application of metaheuristic optimization ensuring stability; Significant reduction of network parameters	Computational overhead associated with metaheuristic optimization algorithms	Long-sequence temperature drift modeling requiring high stability
Parallel Processing Architectures	Parallel architecture combining VMD, MOPSO, and BAS-optimized Elman NN [46]	Parallel separation of denoising and compensation; Prevention of low-frequency motion signal loss	High computational complexity hindering real-time embedded deployment	Signal processing scenarios susceptible to motion signal loss under traditional sequential paradigms
	ICEEMDAN combined with ELM optimized by NSGA-II [47]	Cooperative optimization of high-frequency noise suppression and low-frequency drift compensation utilizing sample entropy classification	Significant processing latency; Highly complex algorithmic structural design	High-precision applications requiring simultaneous random noise reduction and nonlinear drift correction
Physics-Driven and Sensorless Fusion	Geometric nonlinear variational framework [48]	Superposition of thermal displacement fields and nominal vibrations; Provision of genuinely physically interpretable drift predictions	High modeling complexity; Strict reliance on distributed capacitive hardware sensors	Prediction and compensation of frequency drifts and quadrature errors induced by thermal stress
	Closed-loop phase adaptive compensation [49,50]	Online identification and compensation of demodulation phase errors; Complete elimination of external temperature sensors	Limitations to phase-related error sources; Requirement for highly precise circuit control logic	Real-time suppression of scale factor and ZRO drift caused by parasitic capacitance
	MPFC model [51]	Direct extraction and fusion of intrinsic electromechanical control parameters as temperature indicators; Achievement of ultra-low latency sensorless compensation	Requirement for deep access to underlying electromechanical control parameters	Real-time sensorless compensation implemented on FPGAs

**Table 4:** Advanced alignment strategies.

Category	Method	Innovation	Limitations	Application Scenarios
Coarse Alignment	OBA [52,54]	Transformation of alignment into attitude determination via continuous vector observations; Establishment of a globally unified framework for large angular motions	Heavy reliance on external observation vector integrity; Failure of traditional FIR filtering under non-Gaussian impulsive interference	Large angular motions and high-frequency vibration environments
	Adaptive robust estimation [56]	Decoupling of outlier detection from initial attitude via norm invariance; Elimination of time-varying errors utilizing robust parameter identification	Increased algorithmic complexity compared to standard OBA; Requirement for accurate threshold tuning for test statistics	Moving bases subject to non-Gaussian interference and GNSS velocity anomaly jumps
	Analytical self-alignment utilizing dual-position switching [57,58]	Direct suppression of interfering angular rates and accelerations at physical and analytical levels; Provision of data support via information-reusing mechanisms	Fundamental contradiction between required long observation times suppressing MEMS noise and real-time initialization requirements; Restriction to specific RMT INS configurations	Swaying platforms equipped with RMT INS
Fine Alignment	EKF2 and EnPF optimized by KLD [60,61]	Fitting of true posterior probability distributions in non-Gaussian and strongly nonlinear scenarios	Massive computational complexity induced by Jacobian matrix derivations or kernel bandwidth optimization; Susceptibility to system noise amplification	Environments featuring large misalignment angles and strong measurement interference
	Lie group geometric mapping and left-invariant error modeling [65,66]	Elimination of high-order coupling terms via Lie group topological mapping; Proof of global log-linear property achieving robust convergence under extreme misalignment angles	Extremely high mathematical derivation threshold based on differential geometry; Strict adherence requirement to group affine frameworks	Resource-constrained embedded systems facing extreme misalignment angles
	VCKF and multi-channel forward-backward filtering [67,68]	Injection of excitation effects via relative convergence variance lower bounds preventing state desensitization; Enhancement of robustness utilizing historical coarse alignment data	Reliance on heuristic variance lower bounds; Potential delayed processing logic associated with backtracking navigation	Moored swaying conditions exhibiting weakly observable states
	Theoretical analytical modeling under specific constraint conditions [69]	Establishment of explicit analytical mapping relationships between final fine alignment errors and initial IMU attitudes; Identification of *Z*-axis sensors possessing the highest attitude sensitivity	Functionality as a theoretical benchmark rather than a deployable real-time alignment algorithm	Sensor pose planning and a priori error budgeting for moving-base alignment
Multi-Source Aided Alignment	Pseudo-measurement construction utilizing angular velocity and robust estimation [70,71]	Enhancement of heading and vertical channel convergence speeds without additional hardware; Resolution of angular velocity measurement noise correlation utilizing Huber M-estimation	Strong dependence on specific maneuvering trajectories; Limited universality on continuously moving vehicles lacking maneuvering authority	Direct and rapid acquisition under vehicle vibration or non-ideal stationary environments
	Velocity-aided alignment combining trajectory optimization and CGV consistency checks [72,74]	Elimination of coupling between heading errors and velocity biases via 180-degree turn maneuvers; Transformation of DVL outlier detection into geometric consistency evaluation utilizing CGV	Operational constraints requiring specific turning maneuvers; Performance degradation under severe DVL contamination	GNSS-denied environments and extreme sea states missing latitude information
	ST-UKF [77]	Reconstruction of velocity error models replacing specific force terms with gravity terms; Suppression of large MEMS drift under large initial misalignment angles	Increased algorithmic computational complexity associated with unscented transformations	Low-cost sensor performance boundary extension under large initial misalignment angles

**Table 5:** Azimuth gyroscope drift calibration and observability research.

Category	Method	Innovation	Limitations	Application Scenarios
Observability Analysis	State-level OD analysis [78]	Diagnosis of near-zero observability of *Z*-axis drift under moored or stationary conditions	Passive analytical nature lacking constructive solutions for error suppression	Theoretical foundation identifying the necessity of RMT
	PWCS theory combined with TOM and SOM [80]	Establishment of theoretical cornerstones for rotation sequence design	High theoretical complexity requiring specific trajectory segments for full rank	Scientific design of rotation sequences for navigation
	Global observability theory and joint design [81,83,84]	Transition from passive evaluation to active optimization of multiposition and continuous rotation paths	Heavy reliance on high-precision turntables and prolonged calibration cycles exacerbating nonlinear MEMS error coupling	High-precision laboratory or pre-mission system-level calibration
	Adaptive federated filtering utilizing dynamic OD [85]	Transformation of OD into information-sharing factors driving real-time filter adaptation	Dependence on the accuracy of the underlying OD calculation models	Complex time-varying and dynamic environments
Algorithmic Calibration	Augmented KF incorporating horizontal attitude and heading measurements [86]	Enhancement of *Z*-axis drift observability	Restriction to stationary single-axis rotation conditions	Stationary SRINS calibration
	ARKF combining VB and multi-factor robust optimization [88,90]	Suppression of filtering performance degradation and real-time rejection of external DVL outliers	High computational overhead and sensitivity to initial noise priors	Moored conditions and dynamic interference scenarios in complex marine environments
	8-step continuous rotation scheme combined with KF [92]	Excitation and fitting of multiple parameters within ten minutes under non-constant temperature conditions	Limitation to specific temperature drift models	Rapid multi-parameter calibration for micro vehicles
	CFM theory transforming error propagation [95]	Elimination of KF parameter tuning reliance via transformation of complex integral terms into linear least-squares observation matrices	Requirement for buffering massive amounts of historical integral data	Systems rendering manual KF tuning impractical
	Bilinear embedding and adjoint transition matrix theory [96]	Mathematical elimination of nonlinear coupling terms enabling stable convergence under 60° initial attitude errors	Severe computational challenges for onboard MEMS hardware possessing strictly limited resources and single-axis rotation degrees of freedom	Large alignment error scenarios and robust initialization
Mechanical Error Identification	Reciprocating rotation strategy [99]	Identification and physical suppression of unidirectional rotation coupling errors	Neglect of temperature-dependent variations of non-orthogonal angles and internal residual drifts	Suppression of mechanical coupling in single-axis RMT
	Four-position analytical calibration [100]	Quantitative online compensation of heading effects caused by residual drift and electronic Fourier terms	Specificity to periodic error terms rendering it less effective for stochastic drift	Precision online compensation for azimuth alignment
	Periodic averaging combined with stationary physical constraint modeling [97,98]	Isolation of shaft swaying and PID control errors alongside simultaneous convergence of non-orthogonal angles utilizing quadratic temperature models	Confinement primarily to semi-offline preprocessing failing to improve intrinsic weak observability	High-accuracy attitude assurance and static IMU installation misalignment decoupling

**Table 6:** Robust GNSS/SINS integration strategies and methodologies.

Category	Method	Innovation	Limitations	Application Scenarios
Adaptive Filtering	Equivalent VB iteration [63]	Reduction of computational complexity while maintaining adaptive effectiveness in high-dimensional systems	Primary effectiveness confined to slow time-varying noise under Gaussian distribution assumptions	Highly dynamic alignment scenarios prioritizing real-time engineering feasibility
	IMM and HIMM architectures [103,104,105,106]	Integration of kinematic and dynamic models adapting to abrupt velocity changes; Dynamic updating of transition probability matrices adapting to complex hydrological characteristics	Inherent reliance on model set design; Continuous increase in computational load correlating with model quantity	Dynamic vehicle maneuvers and time-varying complex hydrological environments
	Deeply coupled adaptive hybrid frameworks integrating VB, CKF, and IMM [107,108,109]	Parallel processing of time-varying noise estimation and dynamic model switching; Dynamic switching between measurement adaptive modes and hybrid robust modes utilizing runs test hypothesis determination	High algorithmic complexity requiring precise empirical tuning of switching thresholds	Unified suppression of process and measurement errors in shifting noise environments
Robust Estimation	Cascaded iteration incorporating Huber M-estimation and GIMM-KF [112,113]	Adjustment of a priori covariance suppressing process uncertainties; Dual suppression of dynamic model and observation-level outliers	Reliance on empirical tuning of influence functions; Diminished effectiveness against extreme non-Gaussian multipath abrupt changes	Strong maneuvering conditions including severe wave impacts and dual non-Gaussian contamination
	ISSMKF integrating heavy-tailed distributions and VB inference [114,129]	Utilization of Student’s *t*-distributions and adaptive degrees of freedom modeling process and measurement noise; Maintenance of closed-form recursion approximating Gaussian posteriors	Convergence lag susceptibility; Sensitivity to initialization parameters restricting operational stability	Severe heavy-tailed noise contamination and measurement outliers in extreme waters
	ITL criteria combining MCC, MEE, and AMEEF [115,116,117]	Capture of high-order error statistical characteristics; Cooperative suppression of measurement and model anomalies via adaptive kernel bandwidth adjustments and fiducial points	Introduction of highly nonlinear cost functions; Bottlenecks balancing empirical hyperparameter adjustment and real-time computational overhead	Extremely strong non-Gaussian noise and mixed interference in shallow-water and deep-water environments
Multi-Source Fusion	Data-driven generative mechanisms utilizing CEEMD, CNN-LSTM, and AIKF [119,120,121,122]	High-fidelity prediction and self-learning of navigation states maintaining filtering update link continuity during external signal denial	Data-driven black-box fitting lacking physical observability; Divergence during long-term GNSS denial exceeding generalization boundaries	Short-term external signal denial and transient measurement limitation periods
	Marine multi-source tightly coupled augmentation utilizing DVL and USBL [123,124]	Construction of unified observation equations utilizing acoustic beam frequency shifts and raw ranges; Cancellation of common array errors at the physical mechanism level utilizing time backtracking	High engineering costs and heavy reliance on the strict deployment of external beacons	Marine exploration facing insufficient available GNSS observation satellites or limited acoustic beams
	I-EqF leveraging error Lie groups and geometric manifolds [125]	Utilization of Lie group symmetry decoupling error evolution from true trajectories; Significant improvement of global consistency	Extremely high mathematical model derivation thresholds and complex Jacobian matrix computational overhead	Large attitude misalignment angles and covariance distortions generated by severe USV wave swaying
	Heterogeneous sensing tightly coupled architectures incorporating ISIU, GIMM, and RMT [7,113,126,127,128]	Parallel architecture achieving soft cascading of sensors; Reconstruction of heading angle observability in underlying physical dynamics combining single-axis RMT and odometer increments	Introduction of additional hardware complexity and severe cross-sensor scheduling constraints	Complex multi-sensor scheduling and complete GNSS denial scenarios for miniaturized platforms

## Data Availability

The original contributions presented in this study are included in the article. Further inquiries can be directed to the corresponding author.
